# Effect of providing citrus pulp-integrated diet on fecal microbiota and serum and fecal metabolome shifts in crossbred pigs

**DOI:** 10.1038/s41598-023-44741-z

**Published:** 2023-10-16

**Authors:** Dhekra Belloumi, Salvador Calvet, Marta Isabel Roca, Pablo Ferrer, Ana Jiménez-Belenguer, María Cambra-López, Paloma García-Rebollar, Eric Climent, Juan Martínez-Blanch, Marta Tortajada, Empar Chenoll, Almudena Bermejo, Alba Cerisuelo

**Affiliations:** 1 Centro de Investigación y Tecnología Animal, Instituto Valenciano de Investigaciones Agrarias, 12400 Segorbe, Spain; 2 Institute of Animal Science and Technology, Universitat Politècnica de València, 46022 Valencia, Spain; 3 Unidad Analítica, Instituto de Investigación Sanitaria La Fe, 46026 Valencia, Spain; 4 Departamento de Biotecnología, Universitat Politècnica de València, 46022 Valencia, Spain; 5 Departamento de Producción Agraria, ETSIAAB, Universidad Politécnica de Madrid, 28040 Madrid, Spain; 6 ADM Biopolis Sl, 46980 Paterna, Spain; 7 Centro de Citricultura y Producción Vegetal, Instituto Valenciano de Investigaciones Agrarias, 46113 Moncada, Spain

**Keywords:** Biotechnology, Computational biology and bioinformatics, Microbiology, Molecular biology, Molecular medicine

## Abstract

The study aimed to assess the impact of dehydrated citrus pulp (DCP) on growth performance, fecal characteristics, fecal bacterial composition (based on 16S rRNA analysis), and fecal and serum metabolomic profiles in crossbred pigs. 80 finishing pigs Duroc × (Landrace × Large White) were fed either a control diet (C) or a diet with 240 g/kg DCP (T) for six weeks. Including DCP in diets tended to decrease feed intake, increased (p < 0.05) the concentrations of acetic and heptanoic acids and decreased (p < 0.05) fecal butyric and branched-chain fatty acid concentrations in feces. Animals fed DCP exhibited a lower abundance of the genera *Clostridium* and *Romboutsia*, while *Lachnospira* significantly increased. Orthogonal partial least squares discriminant analysis plotted a clear separation of fecal and serum metabolites between groups. The main discriminant fecal metabolites were associated with bacterial protein fermentation and were downregulated in T-fed pigs. In serum, DCP supplementation upregulated metabolites related to protein and fatty acids metabolism. In conclusion, the addition of DCP as an environmentally friendly source of nutrients in pig diets, resulted in modifications of fecal bacterial composition, fermentation patterns, and overall pig metabolism, suggesting improvements in protein metabolism and gut health.

## Introduction

The agro-industry sector generates several by-products that can be used to feed livestock, reducing its dependence on grains and feed costs and contributing to applying a circular economy in the agri-food and livestock sectors. Citrus is one of the most important fruit crops worldwide, with approximately 30% of its yearly harvest being utilized for juice production^1^. Citrus pulp, a by-product of the citrus industry, refers to the solid residue left after squeezing the fruits. It contains peels, seeds, and internal tissues^2^. Dehydrated citrus pulp (DCP) contains a high content of soluble fiber, pectin, and sugar^3^. The DCP is a valuable energy source in pigs’ feeds (boasting digestible energy contents of 13–14 MJ/kg dry matter)^4^ that encourages its use as feed for livestock, increasing the sustainability of the pig sector.

Several studies have shown that DCP can be included in pig diets without altering their growth performance^5,6^, although high inclusion levels can reduce nutrient digestibility^7^. Furthermore, citrus pulp is a relevant source of bioactive compounds, including terpenoids (e.g., limonene) and flavonoids (e.g., hesperidin and naringin)^8^. These molecules and compounds exhibit antioxidant and antibacterial properties^9,10^, improving animal health by inhibiting the growth of pathogenic bacteria^11^.

Gut microbiota plays a crucial role in maintaining the health and metabolism of its host. It has been widely recognized that dietary fiber´s nature and composition are among the most critical factors that can modify microbial populations^12^, considering the specificity of bacteria in fermenting particular substrates^13^. Therefore, manipulating dietary fiber content and source can be a promising approach modulating gut microbiota composition and improving pig health and performance. The fermentation of dietary fiber by gut microbiota produces short-chain fatty acids (SCFAs) such as acetate, propionate, and butyrate. These SCFAs serve as essential energy sources for the host and have been linked to numerous health benefits, including improving gut barrier function, enhancing immune response, and reducing inflammation^14,15^.

A study conducted by Foti et al.^16^ reported that citrus pulp had prebiotic effects since it can selectively stimulate the growth of beneficial bacteria in the gut due to the presence of non-digestible carbohydrates, such as pectin. Moreover, including pectin in weaning pigs´ diet has increased the abundance of *Prevotella*, a microbe associated with fiber degradation, while decreasing the abundance of *Lactobacillus* spp.^17^. Also, Uerlings et al.^18^ found that including 2% citrus pulp in weaning pigs’ feed promoted the growth of *Faecalibacterium* spp. and increased the relative abundance of *Megasphaera* spp., both considered part of the beneficial microbial community.

On the other hand, feces and blood contain a wide array of metabolites that reflect gut microbiota´s composition and metabolic activity and deliver information about the net result of nutrient ingestion, digestion, and absorption, thus providing insights into pig’s health status^19–21^. Recent studies have reported that including citrus pulp in the diet of weaned piglets resulted in modifications in the fermentation patterns in the hindgut by reducing the proteolytic fermentation and increasing the concentrations of some SCFAs^18,22^. Furthermore, previous studies in humans have revealed that consuming orange juice can reduce oxidative stress and inflammatory biomarkers in serum due to its high content of flavanones^23^, suggesting positive effects on health. Therefore, evidence suggests that citrus pulp can positively affect microbiota and metabolism in pigs. However, the number of studies reporting this relationship is still low and mainly focused on young pigs with low DCP inclusion levels in diets. Considering the distinctions in energy partition and metabolism associated with age, the influence of DCP inclusion on microbiota and metabolism in growing-finishing pigs may vary considerably. Additionally, the existing studies contain information on only a few selected metabolites, such as volatile fatty acids (VFA) or branched-chain fatty acids (BCFA), which can limit the interpretation of the results. The application of newly available technologies such as untargeted metabolomics can help to know in more depth the metabolic pathways involved or even discover new bio-markers implicated in the effects of citrus pulp on animals’ health.

The objective of the present study was to investigate the effects of including high levels (240 g/kg) of DCP in growing pigs’ diet on fecal bacterial composition and fecal and serum metabolomic profile, using massive parallel sequencing and untargeted metabolomics to evaluate the relationship among these factors. The results of this study will provide a comprehensive understanding of the impact of this environmentally friendly feeding strategy on gut health and metabolism.

## Results

### DCP and diet composition

As expected, the DCP used in this study showed a high fiber content, especially soluble fiber, and a low crude protein (CP) and fat content (Table 1). Experimental diets had similar levels of CP and energy, but the diet with 240 g/kg of DCP (T) showed a greater amount of fiber and sugars compared with the control diet (C) (Table 2). Regarding the composition of flavonoids in DCP (Table 2), hesperidin was the most abundant, followed by nobiletin and sinensetin. These flavonoids were also identified in the T diet but not in the C diet.

**Table 1:** Analyzed chemical composition of Dehydrated citrus pulp (g/kg DM, unless otherwise specified).

Chemical composition	Dehydrated citrus pulp
Dry matter, g/kg FM^1^	882
Ash	61.4
Gross energy, MJ/kg	17.4
Digestible energy, MJ/kg^2^	13.9
Crude protein	81.0
Ether extract	15.5
NDF^3^	195
ADF^4^	130
ADL^5^	17.9
Total dietary fiber	531
Soluble fiber	336
Sugars	355
Flavonoids, mg/g FM^1^	
Narirutin	1.63
Hesperidin	14.1
Dydimin	0.887
Sinensetin	2.96
Nobiletin	3.09
Tangeretin	0.616

^1^FM: as fed.

^2^Estimated in^4^.

^3^Neutral detergent fiber.

^4^Acid detergent fiber.

^5^Acid detergent lignin.

**Table 2:** Ingredients (g/kg as fed) and nutrient content (g/kg, unless otherwise specified) of the experimental diets.

	Treatments	
	C	T
Ingredients		
Barley	240	0
Corn	200	200
Wheat	350	330
Soybean meal	159	172
Dehydrated citrus pulp	0	240
Palm oil	17.4	30.6
Calcium carbonate	11.9	3.1
Monocalcium phosphate	8.9	11.1
Sodium chloride	3.3	3.0
Sodium bicarbonate	2.0	2.0
Methionine	0.34	0.58
Sulphate l-Lysine	3.48	3.80
l-Threonine	0.54	0.69
Choline chloride	0.60	0.60
Vitamin-mineral premix^1^	3.00	3.00
Analyzed nutrients		
Dry matter, g/kg FM^2^	892	886
Ash	50.6	51.3
Crude protein	180	175
Ether extract	30.7	38.4
NDF^3^	131	141
ADF^4^	33.9	49.3
ADL^5^	3.95	3.90
NDICP^6^	10.9	24.5
ADICP^7^	1.40	2.70
Total dietary fiber	162	220
Soluble fiber	31.2	79.5
Sugar	46.0	97.0
Starch	449	470
Gross energy, MJ/kg	18.4	18.7
Calculated nutrients		
Digestible energy, kcal/kg	3417	3414
Net energy, kcal/kg	2400	2400
Calcium	7.0	7.0
Phosphorus	5.3	5.3
Flavonoids, mg/g FM^2^		
Narirutin	ND^8^	0,449
Hesperidin	ND^8^	4,966
Dydimin	ND^8^	0,234
Sinensetin	ND^8^	0,991
Nobiletin	ND^8^	0,994

*C* 0 g/kg dehydrated citrus pulp, *T* 240 g/kg dehydrated citrus pulp.

^1^Vitamin–mineral premix in the finishing phase provided per kilogram of feed: retinol, 6500 IU (E672); cholecalciferol, 1860 IU (E671); α-tocopherol, 10 mg; menadiones, 0.6 mg; thiamine, 0.8 mg; riboflavin, 3.2 mg; pyridoxin, 1.0 mg; cobalamin, 0.02 mg; niacin, 12 mg; pantothenic acid, 9.60 mg; choline clorure, 116 mg; Fe, 72 mg as FeSO_4_·7H_2_O; Cu, 16 mg as CuSO_4_·5H_2_O; Zn, 80 mg as ZnO; Mn, 40 mg as MnO; I, 1.44 mg as KI and Se, 0.20 mg as Na_2_SeO_3_.

^2^FM: as fed.

^3^Neutral detergent fiber.

^4^Acid detergent fiber.

^5^Acid detergent lignin.

^6^Neutral detergent insoluble crude protein.

^7^Acid detergent insoluble crude protein.

^8^Non-detectable.

### Growth performance and fecal parameters

Results on growth performance and fecal parameters are summarized in Table 3. No statistical differences were observed between the two groups of treatment (C and T) in initial and final body weight (BW), average daily gain (ADG), and feed conversion ratio (FCR) (p > 0.05). However, average daily feed intake (ADFI) tended (p < 0.10) to be lower in pigs fed with the T diet compared to those fed the C diet (3.172 vs. 3.334 kg/days in the T and C diets, respectively). No differences between diets were observed in feces' pH and total ammoniacal nitrogen (TAN) content. In the case of VFA, the total amount (mg/g feces) was similar between treatments, except for the heptanoic acid, which was significantly higher (p < 0.05) in the group of pigs fed the T diet compared to those fed the C diet (0.036 vs. 0.020 mg/g feces in the T and C diets, respectively). However, the VFA profile was markedly different between groups, with pigs fed the T diet showing a higher acetic and heptanoic acid percentage and a lower butyric acid percentage (p < 0.05) than animals fed the C diet (acetic acid: 49.9 vs. 45.8%, heptanoic acid: 0.508 vs. 0.315% and butyric acid: 14.9 vs. 17.3% in the T and C diets, respectively). Additionally, including DCP resulted in a tendency (p < 0.1) for an increase in caproic acid proportion and a reduction in the proportion of isovaleric and isobutyric in feces compared with C-fed animals (caproic acid: 2.46 vs. 1.76%, isovaleric acid: 4.87 vs. 6.08% and isobutyric acid: 2.68 vs. 3.15% in the T and C diets, respectively).

**Table 3:** Effect of dehydrated citrus pulp on pigs' performance and fecal parameters.

Treatment	C	T	SEM	p-value
Initial body weight^1^, kg	71.8	70.8	2.09	0.729
Final body weight^1,2^, kg	128.9	126.8	2.35	0.548
Average daily gain^3^, kg/day	1.156	1.144	0.024	0.718
Average daily feed intake^3^, kg/day	3.334	3.176	0.055	0.061
Feed conversion ratio^3^, g/g	2.85	2.78	0.045	0.302
pH^1^	6.56	6.63	0.104	0.622
Total ammoniacal nitrogen, g/g feces^1^	54.6	51.5	4.07	0.588
Volatile fatty acids, mg/g feces^1^				
Total volatile fatty acids	6.83	6.44	0.509	0.588
Acetic acid	3.27	3.29	0.156	0.927
Propionic acid	1.31	1.24	0.101	0.624
Isobutyric acid	0.205	0.170	0.016	0.135
Butyric acid	1.23	0.991	0.111	0.136
Isovaleric acid	0.395	0.315	0.031	0.082
Valeric acid	0.368	0.311	0.032	0.214
Caproic acid	0.121	0.168	0.022	0.140
Heptanoic acid	0.020	0.036	0.005	0.044
Volatile fatty acids profile, %^1^				
Acetic acid	45.8	49.9	1.028	0.009
Propionic acid	19.7	19.3	0.492	0.571
Isobutyric acid	3.15	2.68	0.192	0.095
Butyric acid	17.3	14.9	0.793	0.036
Isovaleric acid	6.08	4.87	0.441	0.063
Valeric acid	5.27	4.80	0.226	0.148
Caproic acid	1.76	2.46	0.267	0.071
Heptanoic acid	0.315	0.508	0.065	0.041

*C* 0 g/kg dehydrated citrus pulp, *T* 240 g/kg dehydrated citrus pulp.

^1^Measured individually (n = 16).

^2^Covariated by initial values.

^3^Measured by pen (n = 8).

### Fecal bacterial composition

**Figure 1:**
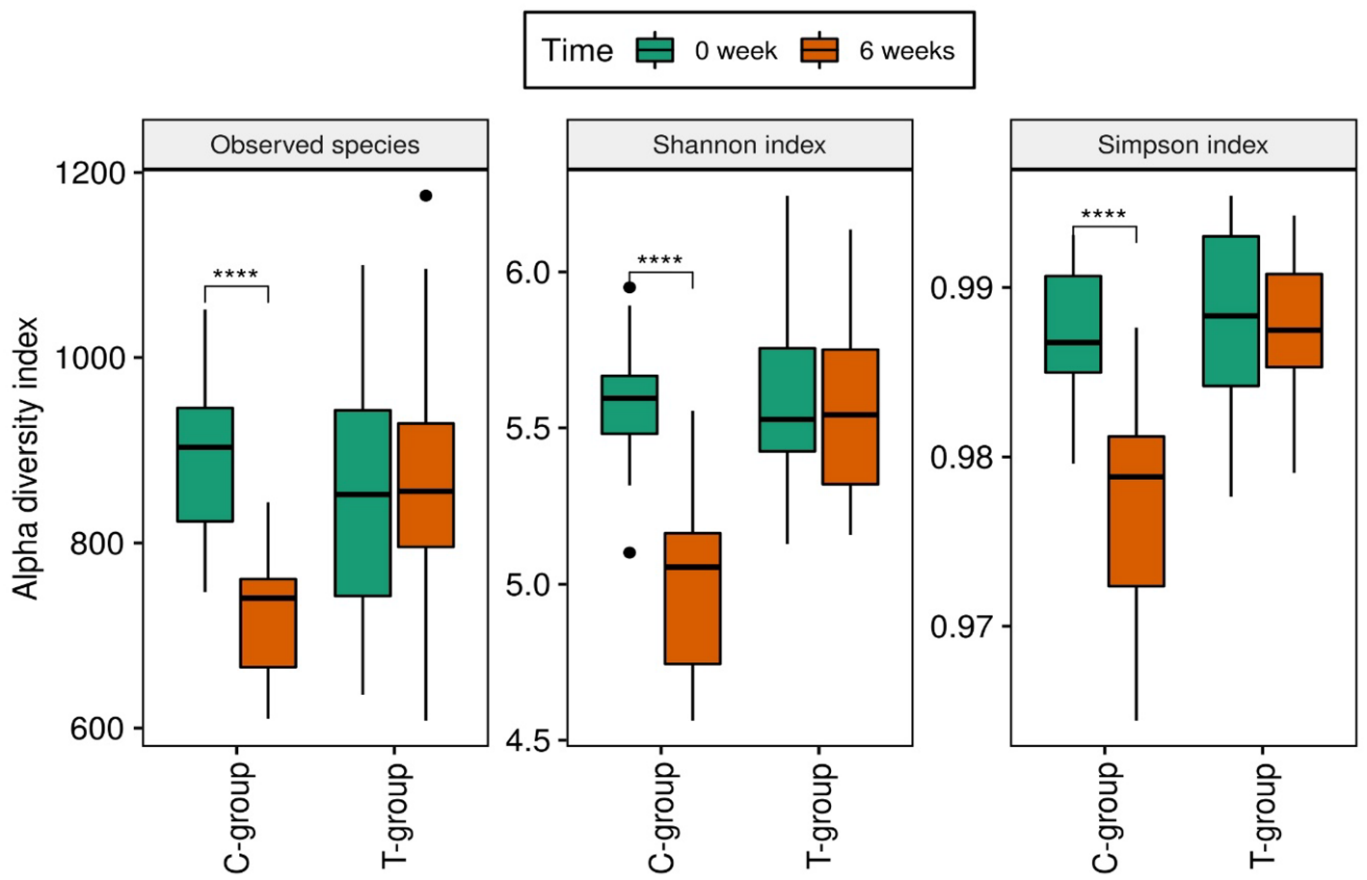
Alpha diversity indexes at basal time (week 0) and after 6 weeks of the experiment. Control group (C-group) and dehydrated citrus-pulp group (T-group). (****p-value < 0.01).

**Figure 2:**
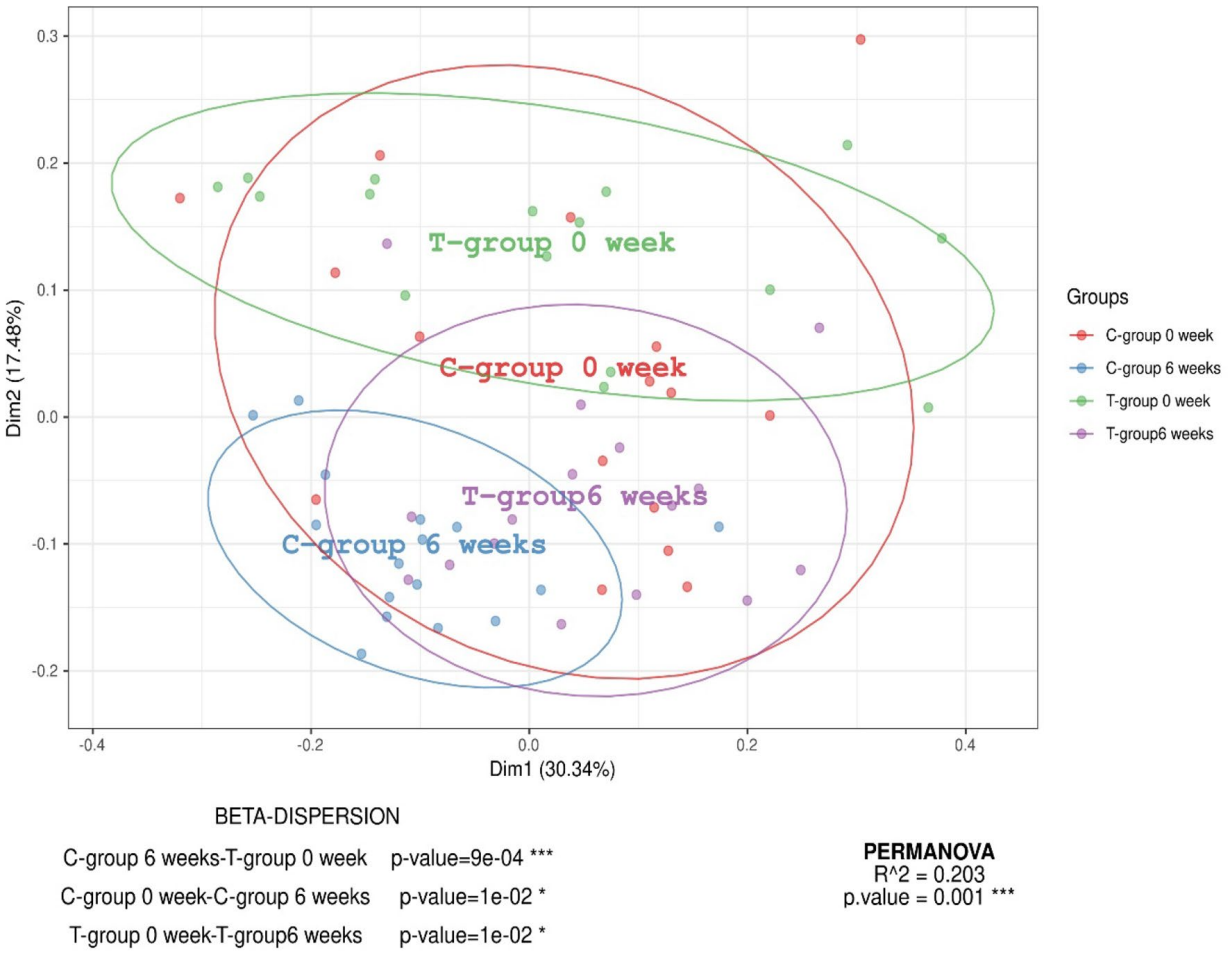
Principal component analysis of the fecal bacterial composition. Comparing the variability of the fecal bacteria composition of control group (C-group) at basal time (week 0, red), citrus-pulp group (T-group) at basal time (week 0, blue), control group (C-group) after 6 weeks of study (green) and citrus-pulp group (T-group) after 6 weeks of study (purple).

**Figure 3:**
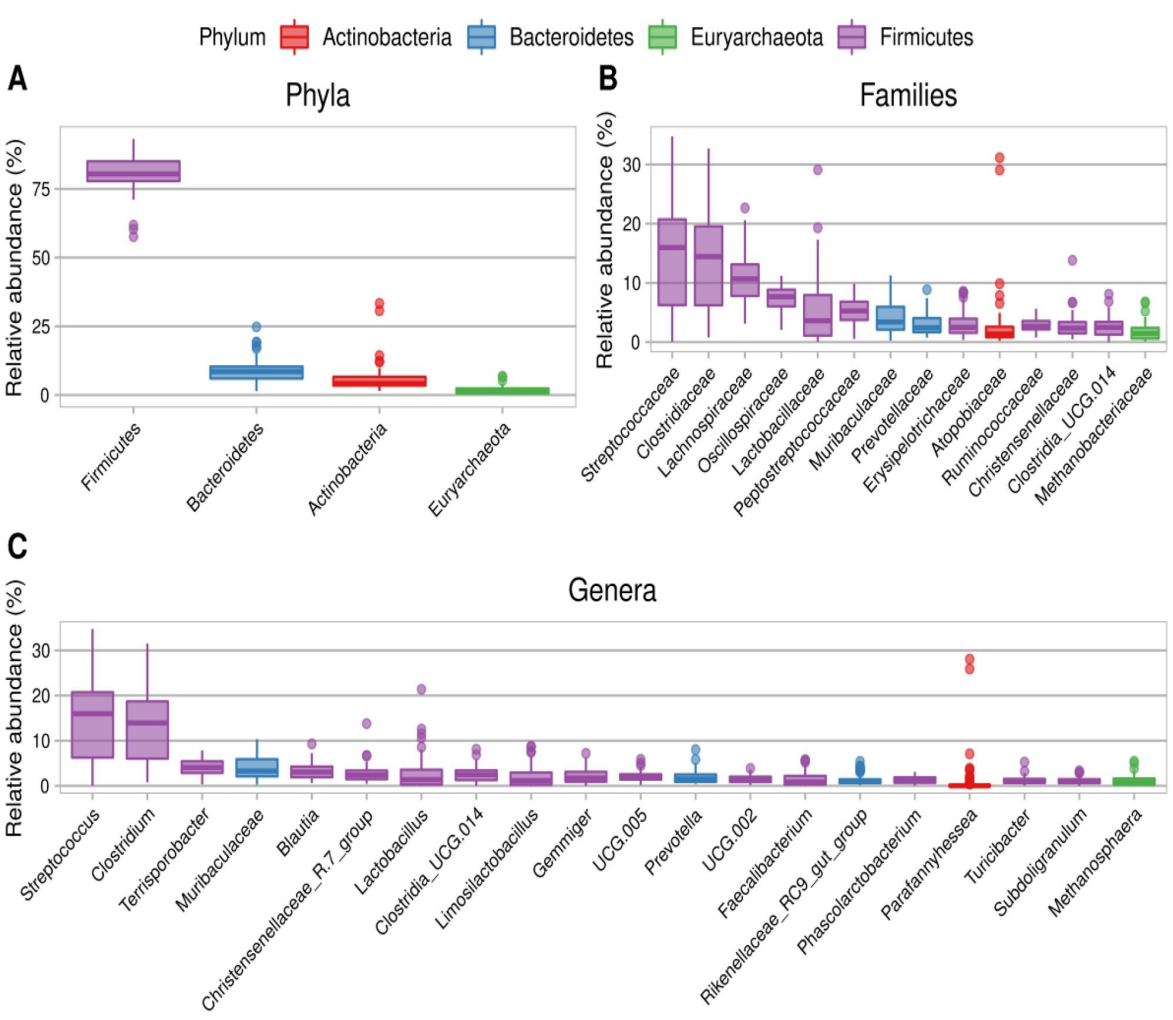
Fecal core bacterial representation (basal time, 0 week); (**A**) Phyla, (**B**) Families, (**C**) Genera.

The fecal bacterial composition was analyzed by massive parallel sequencing, inspecting an average of over 65,358 raw sequences per sample. The number of sequences, average length, total mega bases sequenced, and mean quality per sample can be found in the Supplementary information (Supplementary Table 1). All rarefaction curves showed similar saturation levels, indicating that all samples had been equally covered and could then be compared. The alpha-diversity indexes (Observed species, Shannon and Simpson) analyzed at the basal time (0 week) and after 6 weeks of providing the experimental diets (6 weeks) are presented in Fig. 1. The three diversity indexes decreased significantly (p < 0.001) with time in the C group (from 0 to 6 weeks). However, no differences in alpha-diversity indexes were observed (p > 0.05) between 0 and 6 weeks in the T group. At week 6, Simpson and Shannon indexes were significantly higher (p < 0.001) in the T group than in the C group. Also, the principal component analysis (PCA), a powerful tool for evaluating the beta diversity, demonstrated a separation between C and T groups after 6 weeks of administering the experimental diets (Fig. 2). In the case of bacterial composition, at 0 week (core bacteria), phyla were dominated by *Firmicutes* (reaching 80% of the total), followed by *Bacteroidetes*, *Actinobacteria*, and *Euryarchaeota* (Fig. 3)*.* It is worth highlighting the presence of *Archaea* (in the form of *Euryarchaeota*) in the core microbiota of pigs in the present study. At the family level, *Streptococcaceae* and *Clostridiaceae* showed the highest relative abundances, on average. At the genera level, *Streptococcus* and *Clostridium* were the main genera found, followed by *Terrisporobacter*, an unknown *Muribaculaceae* genus, and *Blautia*.

**Figure 4:**
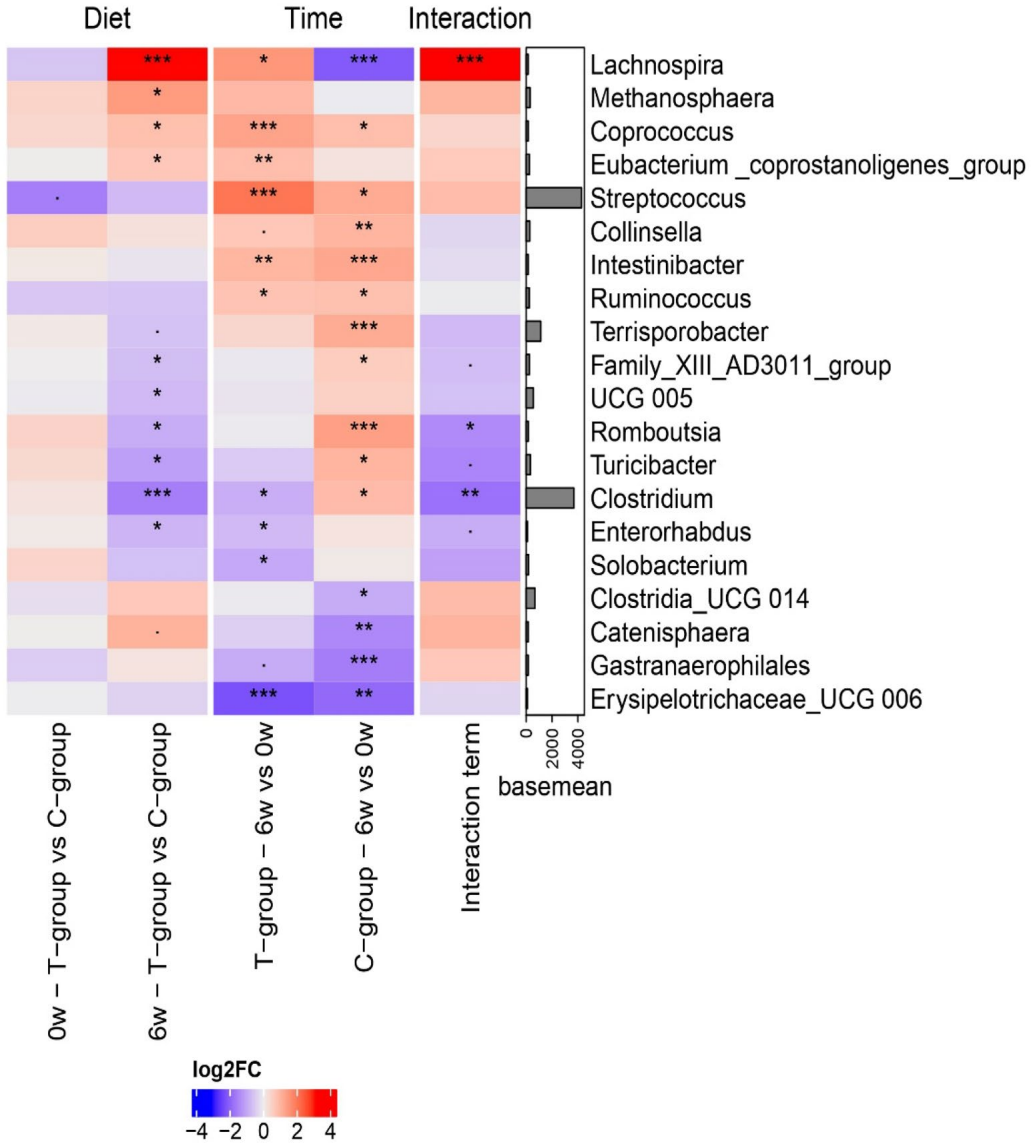
Heatmap summarizing the results of the differential abundance test. The first two columns show the differences between diets at the basal time (0 week) and final time (6 weeks). The third and fourth columns show the temporal changes in each diets. The last column shows the interaction term, which resumes the genera that behave differently between diets over time. In this last column, positive values indicate greater growth in the citrus-pulp group (T-group) over time and a greater decrease in the control group (C-group) or both situations. The p-values are adjusted with false discovery rate (^·^p-value < 0.1, *p-value < 0.05, **p-value < 0.01, ***p-value < 0.001).

The heatmap of the relative bacterial abundances (Fig. 4) shows C and T pigs' fecal bacterial distribution patterns at the genus level according to time, diet, and their interaction (diet × time). At 0 week, no differences were found between treatments regarding bacterial genera. In terms of the evolutionary changes over time (Fig. 4), specific bacterial genera experienced notable shifts in abundance within the T group following 6 weeks of consuming the T diet. *Lachnospira*, *Coprococcus*, *Eubacterium_coprostanoligenes_group*, *Streptococcus*, *Intestinibacter*, and *Ruminococcus* exhibited significantly increased abundances (p < 0.05) after 6 weeks of consuming the T diet. Conversely, the abundances of *Clostridium*, *Enterorhabdus*, *Solobacterium*, and *Erysipelotrichaceae_UCG_006* decreased significantly (p < 0.05) from 0 to 6 weeks of the experiment, indicating a decline in their prevalence within the T group. Similarly, the C group displayed an evolutionary shift, as various genera exhibited significative changes (p < 0.05) in abundance over time. The genera *Coprococcus*, *Streptococcus*, *Collinsella*, *Intestinibacter*, *Ruminococcus*, *Terrisporobacter*, *Family_XIII_AD3011_group*, *Romboutsia*, *Turicibacter*, and *Clostridium* displayed an increase in their respective abundances. In contrast, the abundances of *Lachnospira*, *Clostridia_UCG 014*, *Catenisphaera*, *Gastranaerophilales*, and *Erysipelotrichaceae_UCG 006* decreased significantly (p < 0.05) from week 0 to 6. As a consequence of these changes, after 6 weeks of feeding the experimental diets, the abundances of *Lachnospira*, *Methanosphaera*, *Coprococcus*, and *Eubacterium_coprostanoligenes_group* were significantly higher in the T group compared to the C group, while the genera *Family_XIII_AD3011*, *UCG-005*, *Romboustia*, *Turicibacter*, *Clostridium*, and *Enterorhabdus* were found to be more abundant in C compared with T group, reflecting a response of these genera to the dietary intervention. Regarding the interaction between diet and time, *Lachnospira* demonstrated a significant increase (p < 0.001) over time in the T group while a decrease (p < 0.001) in the C group. Conversely, the abundances of *Romboustia* and *Clostridium* showed a decrease (p < 0.05) in the T group and an increase (p < 0.05) over time in the C group.

### Fecal metabolomic profile

**Figure 5:**
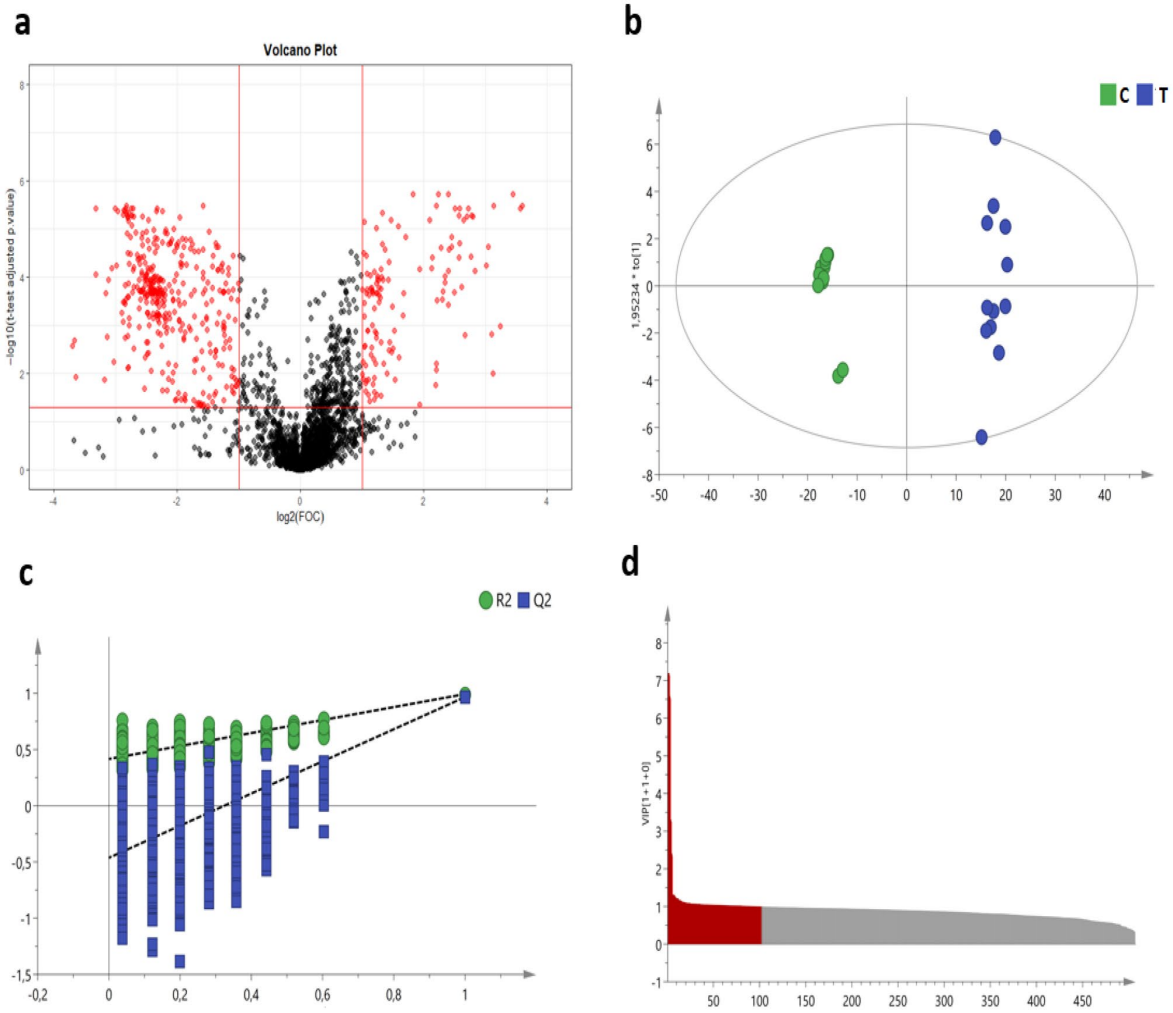
Fecal metabolome analysis. (**a**) Volcano plot; (**b**) OPLS-Da score plot (C: C group, T: T group); (**c**) permutation test plot (R2: model fit of principal component analysis, Q2: predictive ability of the model); (**d**) Features with Variable Importance in the Projection > 1 (in red).

Liquid chromatography-mass spectrometry (LC–MS) based untargeted metabolomics was used to analyze the metabolite composition in the feces of pigs at the end of the experimental period (week 6). After pre-processing and data filtration, 3054 molecular features were selected. After applying the pre-selection of variables through the volcano plot analysis (log2 fold-change (FC) + log10 false discovery rate (FDR) adjusted p-value t-test), 507 molecular features were finally kept for further multivariate analysis. Figure 5 depicts the volcano plot, the orthogonal partial least discriminant analysis (OPLS-DA) score plot, permutation tests, and features with Variable Importance in the Projection (VIP) > 1 obtained for this analysis. The OPLS-DA plot revealed a clear separation between groups, suggesting that dietary inclusion of DCP influenced the main fecal metabolites of pigs. The score plot of the OPLS-DA model showed good parameters (R^2^X = 0.936, R^2^Y = 0.993, Q^2^ = 0.966) and robust cross-validation (CV)-ANOVA (p-value < 0.05) (Fig. 5c). Based on the standard of VIP value above 1, 8 features were selected and tried to be identified as potential biomarkers (Table 4; only putative metabolites with a level of identification equal to 2 are shown in this table). Fecal putative metabolites with a level of identification from 2 to 5 can be found in Supplementary information (Supplementary Table 2).

**Table 4:** Metabolites that most discriminate between C and T groups on fecal metabolomics profile.

Mz	Rt (min)	Putative metabolites	LI	InChI key	Formula	Adduct	FC	Class
204.102	3.78	3-Indolebutyric acid	2	JTEDVYBZBROSJT-UHFFFAOYSA-N	C_12_H_13_NO_2_	M + H	↓	Indoles and derivatives
		Methsuximide	2	AJXPJJZHWIXJCJ-UHFFFAOYSA-N	C_12_H_13_NO_2_	M + H	↓	Pyrrolidines
		Isosalsolidine	2	VBMZFACBWDZUSM-UHFFFAOYSA-N	C_12_H_13_NO_2_	M + H	↓	Isoquinolines and derivatives
256.133	4.34	*N*-Methylschinifoline	2	VBMZFACBWDZUSM-UHFFFAOYSA-N	C_16_H_17_NO_2_	M + H	↑	Quinolines and derivatives
260.090	3.38	Maculosidin	2	SHAVHFJCSQWTFF-UHFFFAOYSA-N	C_14_H_13_NO_4_	M + H	↑	Quinoline alkaloids
		Kokusaginin	2	JBRXRVFXQIKPEA-UHFFFAOYSA-N	C_14_H_13_NO_4_	M + H	↑	Quinoline alkaloids
		Skimmianine	2	SLSIBLKBHNKZTB-UHFFFAOYSA-N	C_14_H_13_NO_4_	M + H	↑	Quinoline alkaloids
263.128	5.62	Dihydrosuberenol	2	YCIWLTLAWPAWSP-UHFFFAOYSA-N	C_15_H_18_O_4_	M + H	↑	Benzenes derivatives
		Dihydrowyerol	2	LQJHQXFOPADMRA-LUAWRHEFSA-N	C_15_H_18_O_4_	M + H	↑	Fatty Acyls
		Enokipodin D	2	KHRRUNIMAKHJPR-UHFFFAOYSA-N	C_15_H_18_O_4_	M + H	↑	Prenol lipids
		Armexifolin	2	QPXLDBMZJNDASA-UHFFFAOYSA-N	C_15_H_18_O_4_	M + H	↑	Prenol lipids
274.154	3.96	(E,E)-Piperlonguminine	2	WHAAPCGHVWVUEX-GGWOSOGESA-N	C_16_H_19_NO_3_	M + H	↑	Benzodioxoles
	4.34	(E,E)-Piperlonguminine	2	WHAAPCGHVWVUEX-GGWOSOGESA-N	C_16_H_19_NO_3_	M + H	↑	Benzodioxoles

*C* 0 g/kg dehydrated citrus pulp, *T* 240 g/kg dehydrated citrus pulp, *mz* Mass-to-charge ratio, *Rt* Retention time, *LI* Level of identification, *InChI Key* IUPAC International Chemical Identifier, *FC* Fold-change of mass peak calculated as the ratio of signal intensity response in T group samples to that in the C group samples: ↑ upregulated (FC > 1), downregulated (FC < 1).

The main putative fecal metabolites affected by including DCP in diets were related to bacterial protein fermentation. These metabolites belonged to classes such as indoles and derivatives (e.g., 3-indolebutyric acid), pyrrolidines (e.g., Methsuximide), isoquinolines (e.g., Isosalsolidine) and their levels were underregulated with the inclusion of DCP. In addition, some specific metabolites found in citrus fruits, such as N-methylschinifoline, maculosidin, kokusaginin, skimmianine, and dihydrosuberenol, increased in the feces of T-fed pigs compared with C pigs.

### Serum metabolomic profile

**Figure 6:**
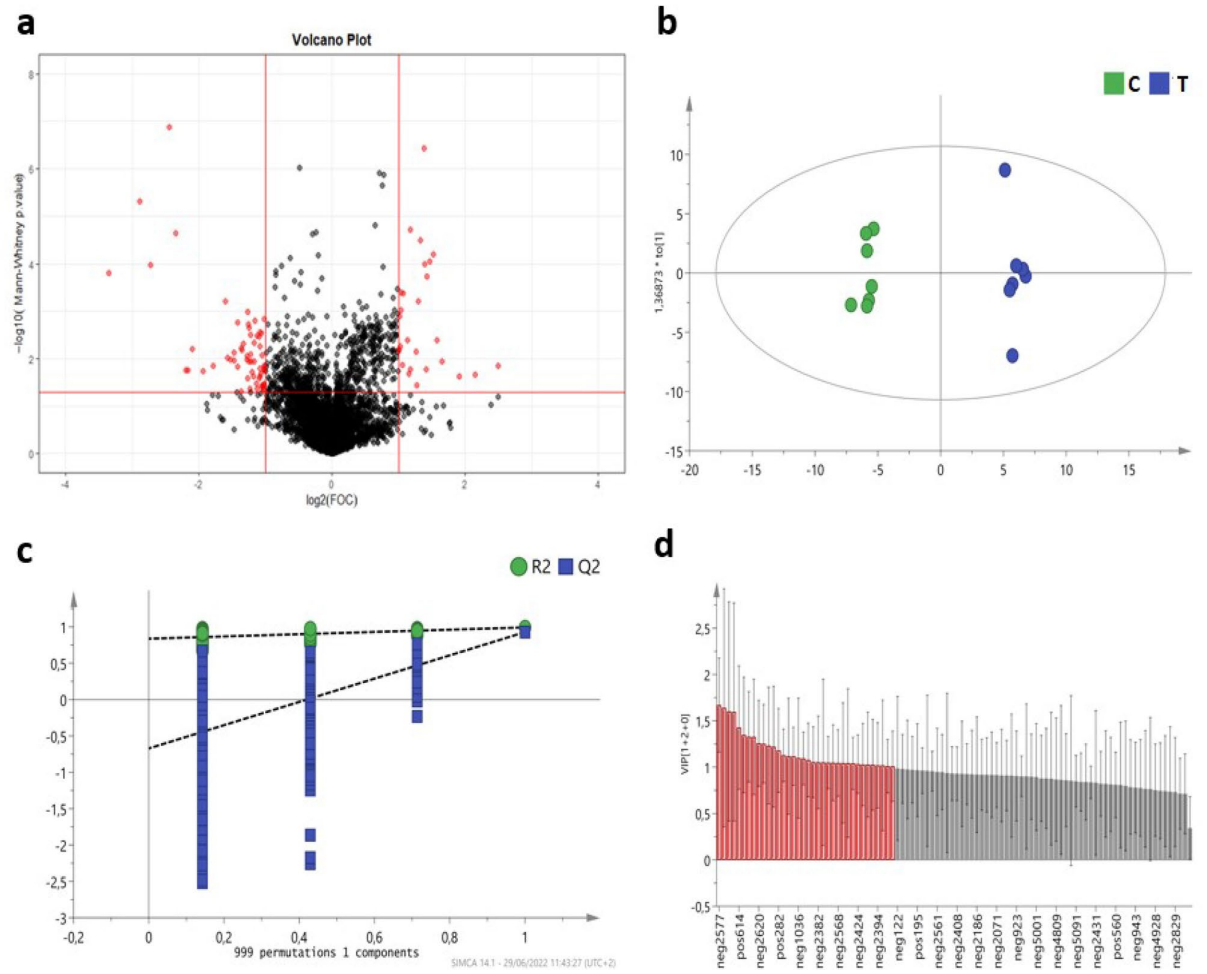
Serum metabolome analysis. (**a**) Volcano plot; (**b**) OPLS-Da score plot (C: C group, T: T group); (**c**) permutation test plot (R2: model fit of principal component analysis, Q2: predictive ability of the model); (**d**) Features with Variable Importance in the Projection > 1 (in red).

The same LC–MS based untargeted metabolomic strategy used for feces was used to analyze the metabolite composition in the serum of pigs at the end of the experimental period (week 6). In this case, 1103 molecular features were selected after pre-processing and filtration of the dataset. After applying the pre-selection of variables through the volcano plot analysis (log2FC + log10 FDR adjusted p-value t-test; Fig. 6a), 96 molecular features were finally kept for further multivariate analysis. The OPLS-DA plot (Fig. 6b), used to identify the differences between the two groups in serum metabolites, revealed that the group fed DCP clustered away from the C group. The OPLS-DA model (Fig. 6b) showed good parameters (R^2^X = 0.754, R^2^Y = 0.992, Q^2^ = 0.928), as well as a validated CV-ANOVA (p-value < 0.05) (Fig. 6c). Based on a threshold of VIP > 1 (Fig. 6d), 36 molecular features were selected and tried to be identified as potential biomarkers (Table 5; only putative metabolites with a level of identification equal to 2 are shown in this table). Serum putative metabolites with a level of identification from 2 to 5 can be found in Supplementary information (Supplementary Table 3).

**Table 5:** Metabolites that most discriminate between C and T groups on serum metabolomics profile.

Mz	Rt (min)	Putative metabolites	LI	InChI key	Formula	Adduct	FC	Class
142.051	1.04	2-Hydroxymethylclavam	2	CRVXTEJTLQIKDI-XINAWCOVSA-N	C_6_H_9_NO_3_	M − H	↑	Alkaloids
		6-Oxopiperidine-2-carboxylic acid	2	FZXCPFJMYOQZCA-UHFFFAOYSA-N	C_6_H_9_NO_3_	M − H	↑	Carboxylic acids and derivatives
		5-ethyl-5-methyl-2,4-oxazolidinedione	2	MGHNWMRNFGYJKE-UHFFFAOYSA-N	C_6_H_9_NO_3_	M − H	↑	Azolidines
		Acrylamide-acrylic acid resin	2	RNIHAPSVIGPAFF-UHFFFAOYSA-N	C_6_H_9_NO_3_	M − H	↑	Carboxylic acids and derivatives
		Trimethadione	2	IRYJRGCIQBGHIV-UHFFFAOYSA-N	C_6_H_9_NO_3_	M − H	↑	Azolidines
		Vinylacetylglycine	2	UKISAGFGRDHYFO-UHFFFAOYSA-N	C_6_H_9_NO_3_	M − H	↑	Carboxylic acids and derivatives
245.049	5.36	[3-(4-methoxyphenyl)propoxy]sulfonic acid	2	LZRUJECSWOAXOK-UHFFFAOYSA-N	C_10_H_14_O_5_S	M − H	↓	Ether
249.022	5.88	4,4′-Sulfonyldiphenol	2	VPWNQTHUCYMVMZ-UHFFFAOYSA-N	C_12_H_10_O_4_S	M − H	↑	Phenols
417.212	6.39	9-Hydroxy-7-megastigmen-3-one glucoside	2	NLPBBNGDXNHAJP-AATRIKPKSA-N	C_19_H_32_O_7_	M + FA − H	↑	Fatty acyls
		Blumenol C glucoside	2	NYLNHNDMNOPWAZ-UHFFFAOYSA-N	C_19_H_32_O_7_	M + FA − H	↑	Carbohydrate derivative
439.195	6.40	Pteroside Z	2	QFXWNTWJLHHEKX-UHFFFAOYSA-N	C_21_H_30_O_7_	M + FA − H	↑	Carbohydrate derivative
		Secoeremopetasitolide B	2	YEMKZDPASIYASW-YFHOEESVSA-N	C_21_H_30_O_7_	M + FA − H	↑	Terpene lactones
444.274	6.62	Uoamine A	2	FYOBRHSIFOGQKX-XROOOXQSSA-N	C_22_H_41_NO_3_S	M + FA − H	↑	Alkaloids
447.259	7.08	*N*-Acetyl-leu-leu-tyr-amide	2	XKHMCCFBVQTJKS-UHFFFAOYSA-N	C_23_H_36_N_4_O_5_	M – H	↑	Peptide
		Sclerotiotide	2	DMCVGIKLKNPCAA-JNPMBWLGSA-N	C_23_H_38_N_4_O_6_	M − H − H_2_O	↑	Oligopeptides
129.066	0.65	(R)-3-Ureidoisobutyrate	2	PHENTZNALBMCQD-GSVOUGTGSA-N	C_5_H_10_N_2_O_3_	M + H − H_2_O	↑	Ureas
		Isoglutammine	2	AEFLONBTGZFSGQ-UHFFFAOYSA-N	C_5_H_10_N_2_O_3_	M + H − H_2_O	↑	Amino acids
		Glycylalanine	2	VPZXBVLAVMBEQI-VKHMYHEASA-N	C_5_H_10_N_2_O_3_	M + H − H_2_O	↑	Oligopeptides
		l-Glutamine	2	ZDXPYRJPNDTMRX-VKHMYHEASA-N	C_5_H_10_N_2_O_3_	M + H − H_2_O	↑	Amino acids
		d-Glutamine	2	ZDXPYRJPNDTMRX-GSVOUGTGSA-N	C_5_H_10_N_2_O_3_	M + H − H_2_O	↑	Amino acids
		Alanylglycine	2	CXISPYVYMQWFLE-VKHMYHEASA-N	C_5_H_10_N_2_O_3_	M + H − H_2_O	↑	Oligopeptides
144.102	0.67	Proline betaine	2	CMUNUTVVOOHQPW-LURJTMIESA-N	C_7_H_13_NO_3_	M + H	↑	Proline and derivatives
		l-2-Amino-3-methylenehexanoic acid	2	ZJMPUKNPMYTOOX-UHFFFAOYSA-N	C_7_H_13_NO_3_	M + H	↑	Alpha amino acids
		3beta,6beta-Dihydroxynortropane	2	MVUIPZFMWQBRCM-UHFFFAOYSA-N	C_7_H_13_NO_3_	M + H	↑	Tropane alkaloids
261.144	3.4	Gamma-Glutamylleucine	2	MYFMARDICOWMQP-YUMQZZPRSA-N	C_11_H_20_N_2_O_5_	M + H	↑	Oligopeptides
275.171	0.6	Lysyl-Glutamine	2	OAPNERBWQWUPTI-YUMQZZPRSA-N	C_11_H_22_N_4_O_4_	M + H	↑	Oligopeptides
		Lysyl-Gamma-glutamate	2	MEFNTMKESSGLSP-UHFFFAOYSA-N	C_11_H_22_N_4_O_4_	M + H	↑	Oligopeptides

*C* 0 g/kg dehydrated citrus pulp, *T* 240 g/kg dehydrated citrus pulp, *mz* Mass-to-charge ratio, *Rt* Retention time, *LI* Level of identification, *FC* Fold-change of mass peak calculated as the ratio of signal intensity response in T group samples to that in the C group samples: ↑ upregulated (FC > 1), downregulated (FC < 1).

All putative metabolites found to be significantly different between groups were upregulated in T-fed pigs compared to C pigs. Most of them were related to the metabolism of protein (e.g., 6-oxopiperidine-2-carboxylic, acrylamide-acrylic acid resin, vinylacetylglycine, *N*-Acetyl-leu-leu-tyr-amide, sclerotiotide, (R)-3-Ureidoisobutyrate, isoglutammine, glycylalanine, l-Glutamine, d-Glutamine, alanylglycine, proline betaine, l-2-Amino-3-methylenehexanoic acid, proline betaine, l-2-Amino-3-methylenehexanoic acid, 3beta,6beta-Dihydroxynortropane, Gamma-Glutamylleucine, Lysyl-Glutamine or Lysyl-Gamma-glutamate) and fatty acids (e.g., [3-(4-methoxyphenyl)propoxy] sulfonic acid, 4,4′-Sulfonyldiphenol, 9-Hydroxy-7-megastigmen-3-one glucoside, Blumenol C glucoside, Pteroside Z or Secoeremopetasitolide B).

## Discussion

In the current study, we investigated the effect of including DCP in pig diets, replacing cereals such as barley and wheat. This strategy contributes to applying a circular economy and reducing the environmental impact of the feed and pigs’ sectors. Additionally, this strategy is a way to reduce competition between feeds and food for humans since DCP (and most of the by-products generated by the agroindustry) is not edible for humans. In pig diets, citrus pulp is a potential energy source due to its high content of sugars and soluble fiber^7,24^. Other studies suggest this ingredient can also benefit gut health in young pigs^18,22^ due to the fiber and the high number of bioactive compounds. In this study, including DCP in diets increased feeds' soluble fiber, sugars, and flavonoid content. The flavonoid profile of the DCP used in the present study, and the T diet contained hesperidin, nobiletin, and sinensetin as the main compounds, as reported by Rangel-Huerta et al.^23^.

Including 240 g/kg of DCP in the T diet, which can be considered a high level of inclusion for this type of ingredient, did not cause significant differences in growth performance between groups of pigs. In previous studies, DCP has been successfully included in fattening pig diets, replacing cereals at maximum levels that range from 50 to 225 g/kg^5,25^. The limits of inclusion in these studies are generally associated with a decrease in feed intake, suggesting that this ingredient can cause satiety or reduce its palatability in pigs at high inclusion levels^6,25^. In the current study, although no significant differences were obtained in feed intake, a tendency was detected for a lower intake in animals from the T group, suggesting that inclusion levels of 240 g/kg DCP may be near the maximum recommended in pigs.

Our results also showed that replacing cereals with DCP impacted VFA but did not affect pH or TAN concentration in feces. The VFA are mainly produced by the bacterial fermentation of dietary fiber or other non-digested nutrients [such as protein or amino acids (AA)] in the hindgut. They provide energy to the host (5–20% of the pig´ energy)^26^ and contribute to maintaining gut integrity^27^. In the present study, the inclusion of DCP in diets increased the acetic acid concentration in feces. This finding was expected, given that acetic acid is the main product of pectin fermentation^5,18^. Heptanoic and caproic acids were also promoted by including DCP in diets. These VFA are medium-chain fatty acids that are valuable energy sources and have a recognized antimicrobial effect^28^.

On the other hand, the tendency for a decrease in isobutyric and isovaleric acids found in pigs fed with DCP has also been previously described in young pigs^5,22^. Isobutyric and isovaleric acids are BCFA generated from proteolysis^29^. Therefore, DCP might have reduced microbial proteolytic fermentation in the hindgut of pigs, as suggested by Uerlings et al.^18^ for weaning pigs. The lower proportion of butyric acid found in the feces of animals from the T group compared with the C group was not expected. Other studies reported greater concentrations of butyric acid in the hindgut of pigs fed 12% of flaxseed meal as a source of soluble fiber^30^, which is positively correlated with gut health since butyric acid promotes energy supply for host metabolism, allows intestinal epithelial cells proliferation and facilitates nutrient absorption. The effects found in VFA concentration and profile when including DCP in diets may be linked to changes in bacterial populations in the gut.

Regarding feces’ bacterial diversity, neither alpha nor beta diversity differed between treatments at week 0. Additionally, no differences were found in the bacteria profile between treatments at baseline (0 week). *Firmicutes* and *Bacteroidetes* were among the main phyla, and *Actinobacteria* was the third phylum found in the fecal core microbiome of pigs. Sutera et al.^31^ and Tardiolo et al.^32^ also found that *Firmicutes* and *Bacteroidetes* were the main phyla in pig feces. According to a meta-analysis that attempted to define a core microbiota in the swine gut^33^, the predominant phyla found in faecal samples were *Firmicutes and Bacteroidetes*, with *Proteobacteria* as a subsequent phylum. In our study, *Proteobacteria* was also detected in all samples but in low amounts (0.45% at 0 week), contrary to the previous study^33^. At the genus level, *Streptococcus* and *Clostridium* were the main genera in the present study, followed by *Terrisporobacter*, an *unknown Muribaculaceae* genus, and *Blautia*. These findings align with the core microbiota defined by Holman et al.^33^, where *Clostridium*, *Lactobacillus*, and *Streptococcus* were among the most abundant genera. The existence of a defined core of bacteria in growing pigs is supported by the meta-study conducted by Holman et al.^33^ and other microbiome studies analyzing the gut microbiome of various pig breeds, which suggests a consistent core microbiome is present in healthy growing pigs. However, interindividual variations and differences in genetics, diet, or farm conditions can be found^34^. After 6 weeks of feeding the experimental diets, the findings concerning the three alpha diversity indexes under investigation revealed a decrease in diversity among the animals in the C group. Conversely, the animals receiving DCP did not exhibit such a decline, indicating the preservation of microbiome diversity. A decrease in microbial diversity is associated with dysbiosis, as highlighted by Le Chatelier et al.^35^. Therefore, it can be inferred that the animals in the C group may be experiencing an imbalance in their gut microbiota composition.

Upon analyzing the beta diversity, a clustering of groups was observed after 6 weeks of treatment. Specifically, there was a separation between animals consuming the diet supplemented with DCP and those consuming the C diet. The observed finding provides evidence of discernible variations in the microbial composition between the two dietary groups. Consequently, this outcome confirms that the inclusion of DCP can modify the faecal microbiota's composition. The substantial concentration of total dietary fiber, soluble fiber, and sugars in DCP suggests that these nutrients may play a crucial role in the observed effect, emphasizing their potential influence on the fecal microbiome of pigs^14,36,37^.

Moreover, another noteworthy aspect of this ingredient is its high content of bioactive compounds, which also possess the potential to exert effects on the microbiota^11^. Specifically, at the end of the experimental period, higher relative abundances of the genera *Lachnospira, Methanosphaera, Coprococcus,* and *Eubacterium_coprostanoligenes_group* were observed in the feces of animals from the T group, in comparison to those of the C group. Notably, these bacterial populations are positively associated with gut health. *Lachnospira*, commonly found in the gut microbiota in pigs, is widely distributed throughout the gastrointestinal tract and has been shown to possess the ability to utilize pectin as a nutrient source^38^. Both *Lachnospira* and *Coprococcus* have been associated with potential anti-inflammatory effects, likely attributed to their ability to produce SCFAs, which have been shown to modulate immune responses and inhibit inflammation^39^. *Methanosphaera* belongs to the *Archaea* domain and is involved in the breakdown of dietary fiber and methane production. Although the abundance of *Archaea* in the digestive tract is relatively low, Deng et al.^40^ and Peng et al.^41^ suggested they are very active and might play important roles in nutrition and metabolism, especially in monogastric animals. *Eubacterium coprostanoligenes_group* are well known for their ability to convert cholesterol to coprostanol^42^. Cholesterol exerts significant physiological effects on animals. Maintaining appropriate cholesterol levels is essential in pigs to ensure optimal health and well-being^43^. The *Eubacterium coprostanoligenes_group*´s metabolic activity helps modulate cholesterol levels in the host and influences its fat metabolism pathways.

On the other hand, the genera *Family_XIII_AD3011_group*, *UCG-005*, *Romboustia*, *Turicibacter*, *Clostridium*, and *Enterorhabdus* were found to be richer in abundance in the C group compared with the T group at the end of the experimental period (6 weeks). While the specific functions and implications of these bacterial groups in the pig gut are not fully understood, some general observations can be made for some of them. *Romboutsia* and *Clostridium* are genera formerly included in the *Clostridia* class*,* associated with carbohydrate degradation^44^ and protein and AA fermentation^45,46^. The congruence between the capacity of these genera for AA fermentation and the fecal metabolite results in this study is highly compelling. Indeed, a significant increase in the abundance of metabolites associated with AA fermentation was observed within group C, further reinforcing the capability of *Romboustia* and *Clostridium* in this metabolic pathway. *Clostridium* is a highly heterogeneous genus that includes both pathogenic and non-pathogenic bacteria. Some of the bacteria in this genus are butyrate-producer bacteria that can promote growth performance^47^. In the present study, the C group of pigs exhibited a greater concentration of butyric acid in feces, consistent with the higher abundance of *Clostridium* in this group. On the other hand, a study conducted by Cisse et al.^48^ reported a negative effect of a diet supplemented with citrus extract and the abundance of pathogen operational taxonomic units belonging to the *Clostridium* genus in sows and piglets. This could explain the reduction in the *Clostridium* abundance and the low fecal butyric acid concentration in the T group of pigs.

On the other hand, *Turicibacter* has been positively correlated with growth performance in pigs^49^. In our study, although there were no significant differences between the two groups in final BW, animals from the C group showed a greater numerical BW (128.9 kg vs 126.8 kg in the C group and T group, respectively) compared with T animals, and the former showed a tendency to a lower feed intake.

Within all the differences found in genera abundances, only three specific genera showed a different evolution with time between treatments (interaction diet × time). These genera were *Lachnospira*, *Romboutsia*, and *Clostridium*. From week 0 to week 6 of the study, *Lachnospira* abundance increased in T-pigs and decreased in C-pigs. However, *Romboutsia* and *Clostridium* both decreased in T-pigs while increased in C-pigs*.* As mentioned above, *Lachnospira* is characterized by inducing anti-inflammatory effects, which ultimately enhance the functioning of the immune system, and *Romboutsia* and *Clostridium* promote carbohydrate and AA fermentation.

Altogether, the outcomes of the present study suggest that the DCP dietary intervention has the potential to positively impact the overall intestinal health of pigs, highlighting the importance of considering pectin and DCP as valuable components in pig nutrition strategies.

Evidence linking gut microbiome with health status has been continuously provided. Studying the metabolomic profile of feces and serum could give some insights into this relationship. The fecal metabolite profile disclosed a series of significant tentative metabolite variations after dietary intervention with DCP as the decrease in the concentrations of 3-indolebutyric acid, methsuximide, and isosalsolidine, belonging to indoles and derivatives, pyrrolidines, and isoquinolines and derivatives classes. These putative metabolites are mainly produced during bacterial protein fermentation. Protein fermentation (or putrefaction) is an anaerobic degradation of non-digested proteins and AA that escape the digestion in the small intestine by the anaerobic bacteria of the hindgut^50,51^. The main metabolites produced by this fermentation are BCFA, ammonia, biogenic amines, phenols, and indoles^46,52^. These metabolites have been associated with impaired gut health^53^ and increasing enteric disease and pathogens^37,54^. In general, protein fermentation occurs when diets are rich in protein since high protein intake may increase the flow of undigested or endogenous protein into the distal parts, hence their fermentation by bacteria^51^. However, protein fermentation may also occur in low-fiber diets containing low fermentable carbohydrates^54^. Thus, in agreement with our results, increasing fiber intake and supplementing diets with fermentable carbohydrates can reduce protein fermentation and its resulting products^55^. The relationship between dietary fiber and protein fermentation may be due to changes in gut microbiota composition promoted by fiber. Additionally, citrus products contain a high concentration of bioactive compounds, such as flavonoids, that have antimicrobial activity against some pathogens^56^ and the potential to modify gut microbiota composition. In our study, the lower concentration of protein fermentation metabolites in the T group animals was due to changes in the microbiota, specifically the reduced abundance of *Clostridium* and *Romboutsia*. These bacteria belong to the *Clostridia* class, known for AA fermentation and deamination, particularly of lysine and proline^57^.

Additionally, in the present study, dietary DCP intake increased the amount of the metabolites N-methylschinifoline, masculosidin, kokusaginin, skimmianine, dihydrosuberenol, dihyrowyerol, enokipodin D, armexifolin, and (E,E)-piperlonguminine in feces. Among these putative metabolites, N-methylschinifoline, masculosidin, kokusaginin, and skimmianine are aromatic heteropoly-cyclic compounds detected in plants belonging to the Rutaceae family (citrus family)^58^. Dihydrosuberenol is a polycyclic compound containing a 1-benzopyran moiety in citrus peel oil. These metabolites could be potential fecal biomarkers of the consumption of citrus fruits^59^. On the other hand, skimmianine has been associated with antibacterial and antimicrobial activities^60^, and dihydrosuberenol, such as other metabolites belonging to the coumarin and derivatives class, is characterized by anti-bacterial, antiviral, and anti-inflammatory activities^61^. Likewise, masculosidin, enokipodin D, and (E,E)-piperlonguminine are also reported as anti-microbial natural dietary metabolites^62,63^. Taking together the results from the fecal bacterial composition and the fecal metabolomic profile suggests that including DCP in the pig diet produces changes in fecal bacterial composition focused on a reduction of protein fermenting bacteria that led to changes in the metabolic profile through a low amount of protein fermentation metabolites. Additionally, DCP induces the presence of some specific metabolites in feces that might reduce the abundance of harmful bacteria, such as Clostridium and increase the abundance of beneficial bacteria.

Regarding serum metabolites, studies showing the effects of citrus pulp on serum metabolome in pigs are scarce in the literature. The results obtained in the present study showed that most of the metabolites affected by the experimental treatments were related to the metabolism of protein and fatty acids. All these metabolites were upregulated in animals from the T group, suggesting that animals from the C group had lower blood serum concentrations of peptides, oligopeptides, AA, proline and derivatives, and alpha AA available for the different functions of the organism compared with animals from the T group. These results agree with the metabolome profile found in feces (downregulation of the metabolites related to protein fermentation in T-pigs) and, thus, a suggested improvement of protein digestion and absorption in the large intestine of T-pigs with lower amounts of residual peptides and AA in the hindgut and a higher amount of peptides and derivates in the bloodstream, compared with C-pigs.

In conclusion, our findings demonstrate that including 240 g/kg DCP in fattening pig diets had a positive impact on fecal bacterial composition by promoting the growth of beneficial bacteria and reducing the abundance of harmful bacteria and residual protein fermentation metabolites. Fecal and serum metabolome profiles suggested that this effect can be attributed to the increased absorption of AA and nitrogen before the hindgut, the potential prebiotic effects of fiber and the potential antibacterial effects of the bioactive components in DCP that reach the feces. Thus, including DCP in pig diets could positively impact gut health, besides being an environmentally friendly animal-feeding practice.

## Methods

All experimental procedures and methods were approved by the Universitat Politècnica de València Ethics Committee (with the registration number 2016/VSC/PEA/00024) and performed in accordance with the current Spanish legislation and guidelines for the care of animals during their use, transportation, experimentation, and sacrifice (Law 32/2007, and its modification 6/2013) and for the protection of animals used in experimentation and other scientific purposes, including teaching (RD 53/2013).

### Animals, diet, and experimental design

A total of 80 male pigs, Duroc-Danbred × (Landrace × Large White), were used in the study. Animals were transferred to our experimental facilities at an initial mean BW of 25.3 ± 3.0 kg. According to their BW (similar BW), animals were assigned to 16 pens (1.54 × 2.88 m^2^) distributed in 2 rooms (8 pens per room) at a rate of 5 animals per pen. Pens were provided with metal ropes as an enrichment material. Before the beginning of the experiment, animals were phase-fed two common commercial feeds (phase 1: from 25 to 35 kg BW, and phase 2: from 35 to 70 kg BW). At 71.2 ± 7.32 kg, pens were randomly assigned to one of two treatments that received two different experimental feeds: Control diet (C), with no DCP inclusion and DCP diet (T), with 240 g/kg of DCP. The DCP was provided by a local fruit juice producer (Zuvamesa, Sagunto, Spain). Control feed was formulated with cereals (barley, corn, and wheat) and soybean meal to meet the requirements of growing-finishing pigs. DCP was included in diet T by substituting barley and small amounts of wheat. Both diets were formulated to be isonutritive. Feeds were provided in pelleted form. The chemical composition of DCP and the ingredient and nutrient composition of the experimental diets, including the main metabolites of the flavonoid profile, are summarized in Tables 4 and 5, respectively. The experiment lasted 6 weeks, in which animals were fed ad libitum (feed was provided with unrestricted access), and water was constantly available throughout the experiment. The environmental air temperature was maintained during the experiment at − 0 to 25 °C.

### Chemical composition and flavonoids concentrations in DCP and experimental diets

The experimental feeds (C and T) and DCP were analyzed for dry matter, ash, starch, total dietary fiber, and ether extract according to the Association of Official Analytical Chemists^64^ procedures. Total sugars were analyzed according to the method of Luff-Schoorl^65^. Different fiber fractions known as neutral detergent fiber (NDF), acid detergent fiber (ADF), and acid detergent lignin concentrations were determined sequentially according to the Van Soest procedure^66^. The contents in soluble fiber were estimated from the difference between total dietary fiber and NDF corrected by CP content in the residue. The gross energy concentration was measured using an isoperibol bomb calorimeter (Parr 6400, Parr Instruments Co., Moline, IL, USA). Total nitrogen was measured by combustion using Leco equipment (model FP-528, Leco Corporation, St. Joseph, MI, USA), and CP was estimated as nitrogen content × 6.25. The CP content in NDF and ADF residues was determined following the standardized procedures in Licitra et al.^67^.

Flavonoid concentrations in samples of DCP and experimental feeds were extracted following a previously described procedure by Cano and Bermejo^68^, with some modifications to adapt the method to a microliter format^69^. Briefly, 50 mg of powdered and dried samples were homogenized in 1 mL of dimethyl sulfoxide/methanol (1:1, v/v). Then the mixture was centrifuged at 4 °C for 30 min at 18,514 rcf. The supernatant was filtered through a 0.45 µm nylon filter and passed to the chromatography vial for Ultra High-Performance Liquid Chromatography coupled to Triple-Quadrupole Mass Spectrometry analysis (UHPLC-QqQ-MS). Ultra-Performance Liquid Chromatography (UPLC)-mass spectrometry analysis of flavonoid concentration was performed on a Thermo Scientific system equipped with a Vanquish separation modules coupled to a TSQ Fortis triple quadrupole mass spectrometer (Thermo Fisher Scientific, Madrid, Spain), using a Luna-Omega PS C18 (100 × 2.1 mm, 1.6 µm, Phenomex) column. The gradient elution program included solvent A (acetonitrile) and solvent B (0.6% acetic acid in water) under the following conditions: initial condition of 5% A for 1 min, which reached 75% A in the next 8 min, then back to the initial condition during 1 min, and held for 5 min (total run time was 15 min). The flow rate was 0.3 mL/min, and the injection volume was 5 µL. The column temperature was maintained at 30 °C, and the autosampler was kept at 4 °C. Chromatograms were recorded at the absorbance of 200–400 nm. Mass analysis was performed in a full scan from 150 to 900 m/z, with an electrospray ionization source in both positive and negative modes. Chromeleon, 7.3 chromatography data system software, was used for data treatment. Compounds were identified by comparing their retention times, UV–Vis spectra, and mass spectral data with authentic standards. Concentrations were determined using an external calibration curve with the flavanones narirutin (RT = 6.54 min, [MH] + 581 m/z), hesperidin (RT = 6.72 min, [MH] + 611 m/z), and didymin (RT = 7.43 min, [MH] + 595 m/z); and the polymethoxyflavones sinensetin (RT = 9.05 min, [MH] + 373 m/z), nobiletin (RT = 9.36 min, [MH] + 403 m/z) and tangeretin (RT = 9.76 min, [MH] + 373 m/z). Narirutin was sourced from Extrasynthese (Genay, France), hesperidin was obtained from Sigma (Barcelona, Spain), and didymin, sinensetin, nobiletin, and tangeretin were purchased from ChromaDex (Irvine, CA, USA). All the solvents used were of UHLC-mass spectrometry grade. Three replicates per sample were analyzed.

### Growth performance measurements

The BW of each animal was measured at the beginning and the end of the experimental period. Additionally, the feed intake per pen was monitored and calculated by subtracting the feed remaining in the feeder on the last day of the study from the total amount of feed offered during the experimental period. The ADG, ADFI and FCR were calculated as well.

### Sample collection

Fecal samples were obtained from a total of 32 animals (16 animals/treatment; 2 animals/pen) after 6 weeks of providing the experimental feeds (end of the trial). These samples were used to measure the pH, VFA concentration, TAN, as well as to perform the bacterial fecal composition and fecal metabolome analysis. Fecal samples for bacterial composition analysis were also collected before the experimental feed administration (week 0). Feces were collected aseptically via rectal stimulation and immediately frozen with liquid nitrogen. For the fecal metabolome analysis, samples were transferred to sterile petri dishes and kept in dry ice until completely frozen. All samples were subsequently stored at − 80 °C until analyses. For the serum metabolome analysis, blood samples from 12 animals (6 animals/treatment, randomly chosen) were collected at slaughter (end of the trial) during exsanguination. During Slaughter, animals were bled by directly sectioning the jugular vein. Approximately 2–3 h after collection, samples were centrifuged at 3500 rpm for 15 min to obtain serum, which was stored in individual aliquots at – 80 °C until analysis.

### Fecal pH, VFA, and TAN

The pH of the fecal samples was measured using a glass electrode (Crison Basic 20+, Crison, Barcelona, Spain). The VFA profile was analyzed with gas chromatography as described by Jouany^70^. Briefly, the samples were diluted with distilled water (1:4) and centrifuged at 3500 rpm for 20 min. Subsequently, 0.9 mL of the resulting supernatant from each sample was mixed with 0.1 mL of a standard (4-methylvaleric). For the TAN analysis, samples were diluted with distilled water (1:7) and centrifuged at 3500 rpm for 10 min. The supernatant of each sample (10 mL) was then taken, acidified to avoid nitrogen volatilization, and then steam distilled using an automatic analyzer (2300 Kjeltec, Foss Analytical, Hilleroed, Denmark).

### Fecal bacterial characterization by 16s rRNA-amplicon sequencing

Genomic DNA of samples was extracted according to the method reported by Yuan et al.^71^ and adding bead beating and enzymatic lysis steps prior to extraction to avoid bias in DNA purification toward the misrepresentation of gram-positive bacteria, with the aid of Magna Pure Compact System (Roche Life Science). Bacterial composition was evaluated by massive parallel sequencing of the hypervariable region V3–V4 of the bacterial 16s rRNA gene. Amplification was run with the S-D-Bact-0341-b-S-17, 5′-CCTACGGGNGGCWGCAG-3′, and the S-D-Bact-0785-a-A-21, 5′-GACTACHVGGGTATCTAATCC-3′ eubacterial primers^72^ and the sequence obtained by MiSeq Illumina Platform in a 300 bp paired-end-run, following the Illumina recommendations. The resulting sequences were split per sample.

R1 and R2 reads were overlapped by PEAR program version 0.9.1^73^ with an overlap of 50 nts and a minimum quality of Q20, providing a single FASTQ file for each sample. The PCR primers were trimmed from the sequences using cutadapt v2.6^74^, and sequences with quality scores under Q20 in the Phred scale were removed with the bbmap software tool reformat (Bushnell, Brian 2104). Amplicon sequence variants (ASVs) were created and denoised using DADA2 package^75^, and chimeras were identified individually in each sample and removed with a consensus decision. The ASVs were annotated using BLAST and the 16S microbial database from NCBI. Sequences that obtained less than 97% of identity against the NCBI database were reannotated using the SILVA database (SILVA 138).

### Fecal and serum untargeted metabolomic analysis using UPLC

#### Sample preparation and metabolomic analyses

Fecal samples were freeze-dried before extraction, as reported by Roca et al.^76^, and stored at − 80 °C until analyses. Then, 0.5 g of each sample was dissolved in 3 mL methanol, homogenized with vortex, and centrifuged (13,000×*g* for 10 min at 4 °C). Regarding serum samples, these were thawed, and 150 µL of cold acetonitrile (0.1%, v/v) was added to 50 µL of serum. The mixture was vortexed, incubated for 3 min at − 20 °C, and centrifuged (13,000×*g* for 10 min at 4 °C). After centrifugation, the supernatants of both types of samples (feces and serum) were collected into aliquots (150 µL) and filtered with Millipore Eppendorf (0.22 µm). Then, 10 µL of the supernatant obtained was transferred to a 96-well plate for LC-QTOF-6550 analysis. In each sample, 90 µL of H_2_O (0.1% HCOOH, v/v) and 10 µL of internal standard mix solution (MIX STDI) (reserpine, leucine, enkephaline, phenylalanine-d5, 20 µM each one) were added. Quality control samples (QCs) were prepared by combining 10 µL from each extract. Blank samples, prepared to replace the extract with ultrapure water, were used to identify artifacts from reagents, the tube, and other materials. Finally, samples, QCs, and blank samples were injected randomly into the chromatographic system. To monitor the stabilities of the instrumental system and the instrumental drift, QCs samples were injected at every 7th sample in each sequence, and the blank samples were performed at the end of the sequence.

The metabolomic analysis was performed using an UPLC system coupled to an iFunnel Q-ToF Agilent 6550 mass spectrometer (Agilent Technologies, CA, USA) with a LC BEH C18 (100 × 2.1 mm, 1.7 µm, Waters, Wexford, Ireland) column from Waters (Wexford, Ireland). The mobile phase was composed of solvent A (0.1% formic acid in water) and solvent B (0.1% formic acid in acetonitrile). The gradient elution was as follows: 98% A (0–1 min), 75% A (1–2 min), 50% A (2–3 min), and 5% A (3–14 min). A 95% mobile phase B was maintained for 3 min, and then a 0.55 min gradient was to return to the initial conditions. The flow rate was set at 400 µL/min with columned temperature maintained at 40 °C. The injection volume was 5 µL, and the autosampler was kept at 4 °C to increase sample stability. The mass spectrometer was a full scan from 50 to 1700 m/z for MS with a scan of 6 Hz collected both in positive (ESI+) and negative (ESI−) electrospray ionization modes. The settings of the electrospray ion source were set as follows: gas temperature: 200 °C; drying gas: 14 L/min; nebulizer: 60 psi; sheath gas temperature: 350 °C; sheath gas flow: 11 L/min. Automatic MS spectra recalibration was carried out by introducing a reference standard containing m/z 149.0233, m/z 121.0508, and m/z 922.0097 into the source via a reference sprayer valve during the analysis. QC sample was also repeatedly analyzed under auto MS/MS, and All-ion (MSE) fragmentation modes at low and high collision energies, which provides valuable information on the (de)protonated molecules and main fragment ions for the identification of discovered metabolites, providing an increased level of confidence in the metabolite annotations.

#### Metabolomic data processing

Raw metabolomic databases were converted to mzXML format using ProteoWizard (http://proteowizard.sourceforage.net/). Then, a series of pre-processing operations were done using an in-house R (v.3.6.1) script with XCMS^77^ and CAMERA^78^ packages, including peak detection, noise filtering, peak alignment, and peak correspondence. A two-dimensional data matrix containing information on molecular features (retention time and m/z) and peak intensities across the samples was generated. After checking for quality data by IS evaluation, the database was filtered according to the quality assurance criteria of coefficient of variation < 30% in QC samples, the presence of the variable in 60% of the samples in at least one of the compared groups, and peak area ratios of sample to blank > 2. Then, the database was normalized using a QC-based robust locally weighted scatter plot smoothing (LOESS) method. The data obtained from ESI+ and ESI− ionization modes were simultaneously merged into one matrix for statistically treatmeant.

#### Metabolites ‘annotation

Metabolites were first identified by database searching using the online CEU Mass Mediator^79^, which combines the results of the Human Metabolome Database (HMBD) (http://hmdb.ca/), KEGG (http://www.kegg.jp)^80,81^, Metlin (http://metlin.scripps.edu/), LipidMaps (http://www.lipidmaps.org), and others databases, within a mass range accuracy of ± 5 ppm. The livestock Metabolome Database^82^ was also used for annotation. The adducts included in the analysis were [M + H] and [M + Na] for ESI+ ionization mode and [M − H] and [M + HCOOH − H] for ESI− ionization mode. Neutral loss by dehydration was also included to identify metabolites for both modes. Sirius^83^ and CSI Finger ID^84^ were then used for molecular formula and structure identification using MS and MS/MS spectra. Finally, five identification levels were determined according to the last categorical system based on the Metabolomics Standard Initiative (MSI) published by Schymanski et al.^85^. The metabolites were categorized into (a) confirmed structure by reference standard (level 1); (b) probably structure (level 2) by library spectrum match (accurate mass and MS/MS spectra); (c) tentative candidates(s) (level 3) if only coincidence with AM was found; (d) unequivocal molecular formula (level 4) and (e) unknown compounds when only exact mass of interest was known but with no match in any database (level 5).

### Statistical analysis

Data on growth performance and fecal parameters were analyzed using the GLM procedure of SAS® (Statistical Analysis System) System Software (Version 9.1, SAS Institute Inc., Cary, North Carolina, EEUU), with the type of diet as the main effect.

Fecal microbiota results were statistically analyzed using R version 4.1.2. ASVs with less than 5 counts were removed from the analysis, and all the ASVs present in only one sample were also removed. The dataset was normalized using the median of ratios method with the poscounts option from the DESeq2 package^86^. Alpha diversity indices (observed species, Simpson, and Shannon) were obtained using the vegan package^87^ and tested between groups using the ANOVA test. Beta diversity was performed by PCA using the factoMine R package^88^ to check whether the samples were grouped into the proposed groups. A heatmap was performed to evaluate changes in fecal bacterial composition with the effect of time, diet, and their interaction as fixed effects using the DESeq2 package^86^.

For the metabolome analyses, statistical analyses were performed using R version 4.1.2 and the software SIMCA 14.1 (Umetrics, Umea, Sweden). A preselection of differential variables was performed with R through a volcano plot, which identifies differential variables using the t-test and FC methods, plotting log2 (FC) on the X-axis against -log10 (p-value) from the t-test on the Y-axis. Those variables with an FC threshold > 2 (or < 0.5) and an FDR-adjusted t-test p-value threshold < 0.05 were finally selected for the following multivariate analysis. For multivariate metabolomic analysis, PCA and OPLS-DA were performed using SIMCA. PCA was carried out to detect patterns in the variable’s matrix and outliers. Then, an OPLS-DA was performed to discriminate between groups (by selecting variables that significantly contributed to the discrimination). The resulting multivariate models were validated by an iterative sevenfold CV approach and 1000 random permutations testing. The validity and robustness of the models were evaluated by R2(Y) (model fit) and Q2(Y) (predictive ability) diagnostics. CV Q2(Y) quality was assessed using the p-value from CV-ANOVA analysis. As a final step, variables were ranked according to their VIP value. Only variables with > 1 and a jack-knife confidence interval that did not include 0 were considered significant contributors to the discrimination and subjected to annotation.

### Ethics declarations

All experimental procedures were approved by the Universitat Politècnica de València Ethics Committee (with the registration number 2016/VSC/PEA/00024). All experiments were carried out following the recommendation in the ARRIVE guidelines (https://arriveguidelines.org/).

### Supplementary Information

Supplementary Tables.

## Data Availability

The sequencing data for this study have been deposited in the European Nucleotide Archive (ENA) at EMBL-EBI under accession number PRJEB57592 (https://www.ebi.ac.uk/ena/browser/view/PRJEB57592). The datasets generated and/or analyzed during the current study are available upon request in the IVIA repository (ReDivia; https://redivia.gva.es) or included in the Supplementary information file of this published article.
