# Supplementary material for: Effect of providing citrus pulp-integrated diet on fecal microbiota and serum and fecal metabolome shifts in crossbred pigs
Source: Sci Rep. 2023 Oct 16;13:17596. doi: 10.1038/s41598-023-44741-z (PMC10579234; doi:10.1038/s41598-023-44741-z)
Supplement: Supplementary file 1 — Supplementary Tables. [file 41598_2023_44741_MOESM1_ESM.pdf]

Supplementary Table 1: Number of sequences, average length, total mega bases (Mb) sequenced, and average quality per sample

Sample	Name	Pair read	Number of sequences	Average length	Total Mb	Average quality
181684	C10	R1	79772	l297,11	23,7	33,49
181684	C10	R2	79772	295,27	23,55	33,3
181685	C10	R1	49751	298,12	14,83	33,74
181685	C10	R2	49751	296,04	14,73	33,32
181686	C10	R1	67717	296,51	20,08	33,44
181686	C10	R2	67717	294,15	19,92	32,89
181687	C10	R1	59600	296,68	17,68	33,77
181687	C10	R2	59600	294,04	17,52	33,11
181688	C15	R1	45586	296,11	13,5	33,53
181688	C15	R2	45586	293,63	13,39	33,03
181689	C15	R1	57712	297,89	17,19	33,8
181689	C15	R2	57712	295,39	17,05	33,19
181690	C15	R1	60163	297,6	17,9	33,51
181690	C15	R2	60163	295,47	17,78	33,14
181691	C15	R1	63757	297,89	18,99	33,79
181691	C15	R2	63757	295,55	18,84	33,25
181692	C16	R1	73637	298,14	21,95	33,74
181692	C16	R2	73637	295,86	21,79	33,26
181693	C16	R1	60014	297,76	17,87	33,78
181693	C16	R2	60014	295,27	17,72	33,26
181694	C16	R1	61771	297,34	18,37	33,72
181694	C16	R2	61771	295,25	18,24	33,37
181695	C16	R1	88202	297,16	26,21	33,82
181695	C16	R2	88202	294,48	25,97	33,18
181696	C26	R1	74739	298,22	22,29	33,76
181696	C26	R2	74739	296,46	22,16	33,64
181697	C26	R1	64373	298,73	19,23	33,95
181697	C26	R2	64373	295,79	19,04	32,96
181698	C26	R1	69208	297,94	20,62	33,83
181698	C26	R2	69208	295,85	20,48	33,58
181699	C26	R1	53954	298,73	16,12	33,95
181699	C26	R2	53954	296,48	16	33,53
181700	C27	R1	51122	298,01	15,24	33,77
181700	C27	R2	51122	296,09	15,14	33,6
181701	C27	R1	57628	298,05	17,18	33,82
181701	C27	R2	57628	295,33	17,02	33,22
181702	C27	R1	56462	297,46	16,79	33,62
181702	C27	R2	56462	295,62	16,69	33,47
181703	C27	R1	55471	298,35	16,55	33,95
181703	C27	R2	55471	295,54	16,39	33,33
181704	C3	R1	54107	297,89	16,12	33,67
181704	C3	R2	54107	295,25	15,97	33,12
181705	C3	R1	55729	298,2	16,62	33,87
181705	C3	R2	55729	295,89	16,49	33,55
181706	C3	R1	67495	298,27	20,13	33,7
181706	C3	R2	67495	296,04	19,98	33,45
181707	C3	R1	86697	298,51	25,88	33,97

181707	C3	R2	86697	296,02	25,66	33,5
181708	C21	R1	72556	296,61	21,52	33,83
181708	C21	R2	72556	294,19	21,35	33,4
181709	C21	R1	58618	298,03	17,47	34,09
181709	C21	R2	58618	295,56	17,32	33,37
181710	C21	R1	68313	297,64	20,33	33,9
181710	C21	R2	68313	295,35	20,18	33,39
181711	C21	R1	64041	298,35	19,11	34,05
181711	C21	R2	64041	295,76	18,94	33,34
181712	C32	R1	63754	296,48	18,9	33,71
181712	C32	R2	63754	294,07	18,75	33,06
181713	C32	R1	72706	298,46	21,7	33,91
181713	C32	R2	72706	295,57	21,49	33,16
181714	C32	R1	49469	298,41	14,76	33,98
181714	C32	R2	49469	296,21	14,65	33,55
181715	C32	R1	53183	298,19	15,86	34,04
181715	C32	R2	53183	295,68	15,72	33,38
181716	C4	R1	56162	298,46	16,76	33,83
181716	C4	R2	56162	296,02	16,63	33,37
181717	C4	R1	59899	298,4	17,87	33,99
181717	C4	R2	59899	295,81	17,72	33,4
181718	C4	R1	71304	297,77	21,23	33,88
181718	C4	R2	71304	295,74	21,09	33,53
181719	C4	R1	63754	298,83	19,05	33,9
181719	C4	R2	63754	296,41	18,9	33,49
181720	C5	R1	72680	298,15	21,67	33,73
181720	C5	R2	72680	295,83	21,5	33,37
181721	C5	R1	52948	297,52	15,75	33,9
181721	C5	R2	52948	294,34	15,58	33,07
181722	C5	R1	62458	297,45	18,58	33,12
181722	C5	R2	62458	296,22	18,5	33,6
181723	C5	R1	57709	298,32	17,22	33,93
181723	C5	R2	57709	296,13	17,09	33,37
181724	C6	R1	49710	297,37	14,78	33,43
181724	C6	R2	49710	295,73	14,7	33,46
181725	C6	R1	62902	297,98	18,74	33,83
181725	C6	R2	62902	294,78	18,54	32,92
181726	C6	R1	64048	297,49	19,05	33,75
181726	C6	R2	64048	295,14	18,9	33,33
181727	C6	R1	54702	297,94	16,3	33,9
181727	C6	R2	54702	294,95	16,13	33,13
181728	C9	R1	64773	298,26	19,32	33,94
181728	C9	R2	64773	295,11	19,11	33,05
181729	C9	R1	65081	298,14	19,4	33,97
181729	C9	R2	65081	295,8	19,25	33,4
181730	C9	R1	68877	297,99	20,52	33,86
181730	C9	R2	68877	295,25	20,34	33,24
181731	C9	R1	85523	298,27	25,51	33,99
181731	C9	R2	85523	295,6	25,28	33,28
181732	C17	R1	84399	297,78	25,13	33,99
181732	C17	R2	84399	295,72	24,96	33,56
181733	C17	R1	63503	298,41	18,95	34,2

181733	C17	R2	63503	295,98	18,8	33,51
181734	C17	R1	71892	297,99	21,42	34,14
181734	C17	R2	71892	295,48	21,24	33,44
181736	C17	R1	67617	297,37	20,11	34,06
181736	C17	R2	67617	294,92	19,94	33,41
181737	C30	R1	75898	297,73	22,6	34
181737	C30	R2	75898	295,15	22,4	33,34
181738	C30	R1	55548	298,47	16,58	34,14
181738	C30	R2	55548	295,57	16,42	33,3
181739	C30	R1	58967	297,2	17,53	34,04
181739	C30	R2	58967	294,7	17,38	33,34
181740	C30	R1	65625	298,29	19,57	34,19
181740	C30	R2	65625	295,71	19,41	33,49
181741	C18	R1	87310	298,37	26,05	34,03
181741	C18	R2	87310	295,95	25,84	33,4
181742	C18	R1	84013	298,65	25,09	34,1
181742	C18	R2	84013	295,96	24,86	33,39
181743	C18	R1	79471	298,57	23,73	34,06
181743	C18	R2	79471	296,51	23,56	33,62
181744	C18	R1	102390	298,5	30,56	34,14
181744	C18	R2	102390	295,72	30,28	33,38
181745	C20	R1	69223	297,77	20,61	33,7
181745	C20	R2	69223	295,97	20,49	33,52
181746	C23	R1	64268	298,43	19,18	33,92
181746	C23	R2	64268	295,67	19	33,25
181747	C23	R1	71792	296,07	21,26	33,66
181747	C23	R2	71792	293,22	21,05	33,01
181748	C23	R1	51186	297,74	15,24	33,85
181748	C23	R2	51186	295,02	15,1	33,27

Supplementary Table 2: Metabolites that discriminate between control (C) and fed with dried citrus pulp (T) groups on fecal metabolomics profile (all identification levels) and the corresponding mz (mass-to-charge-ratio), Rt (retention time), LI (level of identification), InChI Key, formula, adduct, FC (fold change of mass peak calculated as the ratio of signal intensity response in T group samples to that in the C group samples: ↑ upregulated (FC>1), downregulated (FC<1)), and Class (class).

**mz**	**Rt (min)**	**Putative metabolites**	**LI**	**InChI Key**	**formula**	**adduct**	**FC**	**Class**
204,102	3,78	Methsuximide	3	AJXPJJZHWIXJCJ-UHFFFAOYSA-N	C12H13NO2	M+H	↓	-
		Shihunine	3	YWAAZZCPWLHJAN-UHFFFAOYSA-N	C12H13NO2	M+H	↓	-
		3-Indolebutyric acid	2	JTEDVYBZBROSJT-UHFFFAOYSA-N	C12H13NO2	M+H	↓	Indoles and derivatives
		Methsuximide	2	AJXPJJZHWIXJCJ-UHFFFAOYSA-N	C12H13NO2	M+H	↓	Pyrrolidines
		Isosalsolidine	2	VBMZFACBWDZUSM-UHFFFAOYSA-N	C12H13NO2	M+H	↓	Isoquinolines and derivatives
		5-(2-Furanyl)-1,2,3,4,5,6-hexahydro-7H-cyclopenta[b]pyridin-7-one	3	ORZSZFCROJSVII-UHFFFAOYSA-N	C12H13NO2	M+H	↓	-
		ETHYL 3-INDOLEACETATE	3	-	C12H13NO2	M+H	↓	-
		(3-Phenylpropionyl)glycine methyl ester	3	-	C12H15NO3	M+H-H2O	↓	-
		Butylone	3	-	C12H15NO3	M+H-H2O	↓	-
		bk-MDEA	3	-	C12H15NO3	M+H-H2O	↓	-
		bk-MDDMA	3	-	C12H15NO3	M+H-H2O	↓	-
		Carbofuran	3	DUEPRVBVGDRKAG-UHFFFAOYSA-N	C12H15NO3	M+H-H2O	↓	-

		Hydrocotarnine	3	XXANNZJIZQTCBP-UHFFFAOYSA-N	C12H15NO3	M+H-H2O	↓	-
		Metaxalone	3	IMWZZHHPURKASS-UHFFFAOYSA-N	C12H15NO3	M+H-H2O	↓	Phenol ethers
256,133	4,34	N-Methylschinifoline	2	KUJRJLUCKWRGPV-MDZDMXLPSA-N	C16H17NO2	M+H	↑	Quinolines and derivatives
		6-(1,2,3,4-Tetrahydro-6-methoxy-2-naphthyl)-2(1H)-pyridone	3	ZJPDJIYPZYRXEC-UHFFFAOYSA-N	C16H17NO2	M+H	↑	-
		1-Methyl-6-(1,2,3,4-tetrahydro-6-hydroxy-2-naphthyl)-2(1H)-pyridone	3	APBIVLKQBBISBA-UHFFFAOYSA-N	C16H17NO2	M+H	↑	-
		N-benzoyl-L-phenylalaninol	3	-	C16H17NO2	M+H	↑	-
		N-Benzoyl-L-phenyalaninol	3	-	C16H17NO2	M+H	↑	-
		Hexahomomethionine	3	XVGBKWQWYRNGDG-UHFFFAOYSA-N	C11H23NO2S	M+Na	↑	-
		Norgalanthamine	3	AIXQQSTVOSFSMO-RBOXIYTFSA-N	C16H19NO3	M+H-H2O	↑	-
		alpha-Erythroidine	3	IXPDLJKEPLTCOU-QJNGTDTRNA-N	C16H19NO3	M+H-H2O	↑	-
		Sanguinine	3	OYSGWKOGUVOGFQ-RBOXIYTFSA-N	C16H19NO3	M+H-H2O	↑	-
		beta-Erythroidine	3	PXWINCSLFXUWBZ-BBRMVZONSA-N	C16H19NO3	M+H-H2O	↑	-
		(-)-8-Demethylmaritidine	3	TZTBAJFJEZRQCV-RLCCDNCMSA-N	C16H19NO3	M+H-H2O	↑	-
		4'-O-Methylnorbelladine	3	SDLILULALIDNSO-UHFFFAOYSA-N	C16H19NO3	M+H-H2O	↑	-
		Lunacrine	3	FMEKJMQGMONLTQ-CYBMUJFWSA-N	C16H19NO3	M+H-H2O	↑	-

		(E,E)-Piperlonguminine	3	WHAAPCGHVWVUEX-GGWOSOGESA-N	C16H19NO3	M+H-H2O	↑	-
		(2E)-Piperamide-C5:1	3	XZTCTKKANUDQCW-QHHAFSJGSA-N	C16H19NO3	M+H-H2O	↑	-
		(4aR,10bS)-Normaritidine	3	-	C16H19NO3	M+H-H2O	↑	-
260,09	3,38	Clobenzorex	2	LRXXRIXDSAEIOR-UHFFFAOYSA-N	C16H18ClN		↑	Benzene and substituted derivatives
		AG-370	3	-	C15H9N5	M+H	↑	-
		Skimmianine	3	SLSIBLKBHNKZTB-UHFFFAOYSA-N	C14H13NO4	M+H	↑	-
		Maculosidine	3	SHAVHFJCSQWTFF-UHFFFAOYSA-N	C14H13NO4	M+H	↑	-
		Kokusaginine	3	JBRXRVFXQIKPEA-UHFFFAOYSA-N	C14H13NO4	M+H	↑	-
		2,3-dihydro-1,4-benzodioxin-2-ylmethyl dimethylcarbamate	3	-	C12H15NO4	M+Na	↑	-
		3-Hydroxy-carbofuran	3	RHSUJRQZTQNSLL-UHFFFAOYSA-N	C12H15NO4	M+Na	↑	-
		Salsoline-1-carboxylate	3	CJEFWISJWQNPSZ-GFCCVEGCSA-N	C12H15NO4	M+Na	↑	-
		N-lactoyl-Phenylalanine	3	IIRJJZHHNGABMQ-WPRPVWTQSA-N	C12H15NO4	M+Na	↑	-
		3-(3,4-dihydroxyphenyl)-N-(3-oxopropyl)propanimidic acid	3	-	C12H15NO4	M+Na	↑	-
		Isoflavipucine	3	-	C12H15NO4	M+Na	↑	-
		Asp Gly Ser	3	-	C9H15N3O7	M+H-H2O	↑	-

		Gly Ser Asp	3	-	C9H15N3O7	M+H-H2O	↑	-
		Ser Asp Gly	3	-	C9H15N3O7	M+H-H2O	↑	-
		Asp Ser Gly	3	-	C9H15N3O7	M+H-H2O	↑	-
		Ser Gly Asp	3	-	C9H15N3O7	M+H-H2O	↑	-
		Gly Asp Ser	3	-	C9H15N3O7	M+H-H2O	↑	-
		Lycomarasmine B	3	YRSDOJQPYZOCMY-ROLXFIACSA-N	C9H15N3O7	M+H-H2O	↑	-
263,128	5,62	Dihydrosuberenol	2	YCIWLTLAWPAWSP-UHFFFAOYSA-N	C15H18O4	M+H	↑	Benzene and substituted derivatives
		Dihydrowyerol	2	LQJHQXFOPADMRA-LUAWRHEFSA-N	C15H18O4	M+H	↑	Fatty Acyls
		Enokipodin D	2	KHRRUNIMAKHJPR-UHFFFAOYSA-N	C15H18O4	M+H	↑	Prenol lipids
		5,6-Dihydro-4-methoxy-6-[2-(4-methoxyphenyl)ethyl]-2H-pyran-2-one	2	IZGFAKZIDOQLHS-UHFFFAOYSA-N	C15H18O4	M+H	↑	Kavalactones
		Armexifolin	2	QPXLDBMZJNDASA-UHFFFAOYSA-N	C15H18O4	M+H	↑	Prenol lipids
		Sudan Brown RR	3	AHWMWMNEYBHQNL-FMQUCBEESA-N	C16H14N4	M+H	↑	-
		Farinosin	3	CYMAWWCMMZZOTB-KJMJJSJTSA-N	C15H18O4	M+H	↑	-
		Grosshemin	3	YGMIBVIKXJJQQJ-MSOSQAFRSA-N	C15H18O4	M+H	↑	-

		Parthenin	3	LLQCRTZROWMVOL-JISBIHODSA-N	C15H18O4	M+H	↑	-
		Austricine	3	-	C15H18O4	M+H	↑	-
		Epoxy (4,5a)-4,5-Dihydrosantonin	3	-	C15H18O4	M+H	↑	-
		Methylripariochromene A	3	OZQYZUMHJKYJEK-UHFFFAOYSA-N	C15H18O4	M+H	↑	-
		(+)-Marmasmic acid	3	BIUVCPLWWOLECJ-LIHVSQBMSA-N	C15H18O4	M+H	↑	-
		Artemisin	3	LUHMMHZLDLBAKX-DBIGVJDZSA-N	C15H18O4	M+H	↑	-
		Mexicanin I	3	ZVLOPMNVFLSSAA-UEIZXJDVSA-N	C15H18O4	M+H	↑	-
		Stramonin B	3	AYSAVJUPCHRCGO-ODHINMQUSA-N	C15H18O4	M+H	↑	-
		Microhelenin A	3	UAYKGHBWLFRCCG-CBPBZNKLSA-N	C15H18O4	M+H	↑	-
		Helenalin	3	ZVLOPMNVFLSSAA-XEPQRQSNSA-N	C15H18O4	M+H	↑	-
		Dihydrogriesenin	3	QOWDVJAIJZLWBJ-UXOAXIEHSA-N	C15H18O4	M+H	↑	-
		Cyclogregatin	3	-	C15H18O4	M+H	↑	-
		Daldinin B	3	-	C15H18O4	M+H	↑	-
		3-[4-hydroxy-2-methoxy-3-(3-methylbut-2-en-1-yl)phenyl]prop-2-enoic acid	3	-	C15H18O4	M+H	↑	-
		Ustusorane A	3	-	C15H18O4	M+H	↑	-
		Asperanin C	3	-	C15H18O4	M+H	↑	-
		Pentalenolactone E	3	-	C15H18O4	M+H	↑	-
		Imiquimod	3	DOUYETYNHWVLEO-UHFFFAOYSA-N	C14H16N4	M+Na	↑	-

		Autumnolide	3	NWSWVIKHALGAER-KVLFNOHQSA-N	C15H20O5	M+H-H2O	↑	-
		Hymenoflorin	3	-	C15H20O5	M+H-H2O	↑	-
		Betaxolol(deaminated)	3	-	C15H20O5	M+H-H2O	↑	-
		3-[(4-Carboxy-4-methylpentyl)oxy]-4-methylbenzoic acid (Gemfibrozil M3)	3	-	C15H20O5	M+H-H2O	↑	-
		Phaseic acid	3	-	C15H20O5	M+H-H2O	↑	-
		Artabsinolide A	3	-	C15H20O5	M+H-H2O	↑	-
		Vulgarolide	3	-	C15H20O5	M+H-H2O	↑	-
		Nigellic acid	3	-	C15H20O5	M+H-H2O	↑	-
		13-Hydroxyabscisic acid	3	-	C15H20O5	M+H-H2O	↑	-
		8-Deoxy-11,13-dihydroxygrosheimin	3	-	C15H20O5	M+H-H2O	↑	-
		(1beta,4alpha,5alpha,6beta,8alpha,10b)-1,10:4,5-Diepoxy-6-hydroxy-7(11)-germacren-12,8-olide	3	-	C15H20O5	M+H-H2O	↑	-
		Dihydromarasmone	3	-	C15H20O5	M+H-H2O	↑	-
		2-hydroxy-3-[4-hydroxy-3-methoxy-5-(3-methylbut-2-en-1-yl)phenyl]propanoic acid	3	-	C15H20O5	M+H-H2O	↑	-
		Crispolide	3	-	C15H20O5	M+H-H2O	↑	-

		Asperaculin A	3	-	C15H20O5	M+H-H2O	↑	-
		Flakinin A	3	-	C15H20O5	M+H-H2O	↑	-
		(+)-Hirusten-12-oic acid	3	-	C15H20O5	M+H-H2O	↑	-
		(+)-8'-hydroxyabscisic acid	3	-	C15H20O5	M+H-H2O	↑	-
		7'-hydroxyabscisic acid	3	-	C15H20O5	M+H-H2O	↑	-
		(-)-(7R,10S)-10-hydroxysydowic acid	3	-	C15H20O5	M+H-H2O	↑	-
		(1S,6S,7R)-aspergilloid C	3	-	C15H20O5	M+H-H2O	↑	-
		(7R,8R)-1,8-epoxy-11-hydroxy-sydonic acid	3	-	C15H20O5	M+H-H2O	↑	-
		(7S,11R)-12-hydroxy-sydowic acid	3	-	C15H20O5	M+H-H2O	↑	-
		(-)-(7R,10R)-iso-10-hydroxysydowic acid	3	-	C15H20O5	M+H-H2O	↑	-
		(-)-(R)-cyclo-hydroxysydonic acid	3	-	C15H20O5	M+H-H2O	↑	-
		(-)-(7S,8R)-8-hydroxysydowic acid	3	-	C15H20O5	M+H-H2O	↑	-
		2-(5-hydroxy-4-methylpentyl)-2-methylbenzo[d][1,3]dioxole-5-carboxylic acid	3	-	C15H20O5	M+H-H2O	↑	-
		N-Histidyl-2-Aminonaphthalene (ßNA)	3	-	C16H16N4O	M+H-H2O	↑	-

		Hydroxystilbamidine	3	-	C16H16N4O	M+H-H2O	↑	-
274,145	4,34	(E,E)-Piperlonguminine	2	WHAAPCGHVWVUEX-GGWOSOGESA-N	C16H19NO3	M+H	↑	Benzodioxoles
		(4aR,10bS)-Normaritidine	3	-	C16H19NO3	M+H	↑	-
		(2E)-Piperamide-C5:1	3	-	C16H19NO3	M+H	↑	-
		Norgalanthamine	3	-	C16H19NO3	M+H	↑	-
		alpha-Erythroidine	3	-	C16H19NO3	M+H	↑	-
		Sanguinine	3	-	C16H19NO3	M+H	↑	-
		beta-Erythroidine	3	-	C16H19NO3	M+H	↑	-
		(-)-8-Demethylmaritidine	3	-	C16H19NO3	M+H	↑	-
		4'-O-Methylnorbelladine	3	-	C16H19NO3	M+H	↑	-
		Lunacrine	3	-	C16H19NO3	M+H	↑	-
		(S)-Edulinine	3	-	C16H21NO4	M+H-H2O	↑	-
		Phyllalbine	3	-	C16H21NO4	M+H-H2O	↑	-
		Convolvine	3	-	C16H21NO4	M+H-H2O	↑	-
274,144	3,96	(2E)-Piperamide-C5:1	3	-	C16H19NO3	M+H	↑	Benzodioxoles
		(E,E)-Piperlonguminine	2	WHAAPCGHVWVUEX-GGWOSOGESA-N	C16H19NO3	M+H	↑	Benzodioxoles
		(4aR,10bS)-Normaritidine	3	-	C16H19NO3	M+H	↑	-
		Norgalanthamine	3	-	C16H19NO3	M+H	↑	-
		alpha-Erythroidine	3	-	C16H19NO3	M+H	↑	-
		Sanguinine	3	-	C16H19NO3	M+H	↑	-
		beta-Erythroidine	3	-	C16H19NO3	M+H	↑	-

		(-)-8-Demethylmaritidine	3	-	C16H19NO3	M+H	↑	-
		4'-O-Methylnorbelladine	3	-	C16H19NO3	M+H	↑	-
		Lunacrine	3	-	C16H19NO3	M+H	↑	-
		(S)-Edulinine	3	-	C16H21NO4	M+H-H2O	↑	-
		Phyllalbine	3	-	C16H21NO4	M+H-H2O	↑	-
		Convolvine	3	-	C16H21NO4	M+H-H2O	↑	-
362,196	4,54	Phalaenopsine T	3	-	C20H27NO5	M+H	↑	-
405,202	3,4	7-Geranylformononetin	3	-	C26H28O4	M+H	↑	-
		Boesenbergin A	3	-	C26H28O4	M+H	↑	-
		Boesenbergin B	3	-	C26H28O4	M+H	↑	-
		Lys Glu Glu	3	-	C16H28N4O8	M+H	↑	-
		Glu Lys Glu	3	-	C16H28N4O8	M+H	↑	-
		Glu Glu Lys	3	-	C16H28N4O8	M+H	↑	-
		3,12-Dioxochola-1,4,9(11)-trien-24-oic Acid	3	-	C24H30O4	M+Na	↑	-
		Ammoresinol	3	-	C24H30O4	M+Na	↑	-
		Assafoetidin	3	-	C24H30O4	M+Na	↑	-
		Gancaonin R	3	-	C24H30O4	M+Na	↑	-
		(3'alpha,5'alpha,9'xi,10'beta)-O-(3-Hydroxy-7-drimen-11-yl)umbelliferone	3	-	C24H30O4	M+Na	↑	-
		Farnesiferol B	3	-	C24H30O4	M+Na	↑	-
		(-)-Farnesiferol C	3	-	C24H30O4	M+Na	↑	-
		Foetidin	3	-	C24H30O4	M+Na	↑	-
		Farnesiferol A	3	-	C24H30O4	M+Na	↑	-

		Alpha,alpha,6-Trimethyl-4-(2-prenyl-3-methyl-5-hydroxyphenoxy)-2,3-dihydrobenzofuran-2beta-methanol	3	-	C24H30O4	M+Na	↑	-
		Alpha,alpha,6-Trimethyl-4-(3-hydroxy-5-methylphenoxy)-5-prenyl-2,3-dihydrobenzofuran-2beta-methanol	3	-	C24H30O4	M+Na	↑	-
		LysoPA(i-14:0/0:0)	3	-	C17H35O7P	M+Na	↑	-
		PA(14:0/0:0)[U]	3	-	C17H35O7P	M+Na	↑	-
		PA(14:0/0:0)	3	-	C17H35O7P	M+Na	↑	-
		Blasticidin S	3	-	C17H26N8O5	M+H-H2O	↑	-
		Blasticidin S	3	-	C17H26N8O5	M+H-H2O	↑	-
		Cedrelone	3	-	C26H30O5	M+H-H2O	↑	-
		7-Deacetoxy-7-Oxodeoxygedunin	3	-	C26H30O5	M+H-H2O	↑	-
		7-Desacetoxy-6,7-Dehydrogedunin	3	-	C26H30O5	M+H-H2O	↑	-
		5'-Prenylxanthohumol	3	-	C26H30O5	M+H-H2O	↑	-
		1-Methoxyficifolinol	3	-	C26H30O5	M+H-H2O	↑	-
		Kanzonol J	3	-	C26H30O5	M+H-H2O	↑	-
		P-hydroxybenzaldehyde, chevaline B, chevaline C, S14-95	3	-	C26H30O5	M+H-H2O	↑	-
		Arugosin O	3	-	C26H30O5	M+H-H2O	↑	-

		Kushenol R	3	-	C26H30O5	M+H-H2O	↑	-
		Xanthoangelol G	3	-	C26H30O5	M+H-H2O	↑	-
		4-Hydroxyglenoblone	3	-	C26H30O5	M+H-H2O	↑	-
		5-Deoxyhomoflemingin	3	-	C26H30O5	M+H-H2O	↑	-
		1-Methoxyficifolinol	3	-	C26H30O5	M+H-H2O	↑	-
		Kushenol U	3	-	C26H30O5	M+H-H2O	↑	-
		(-)-Linderol A	3	-	C26H30O5	M+H-H2O	↑	-
		Euchrenone a8	3	-	C26H30O5	M+H-H2O	↑	-
		Alopecurone G	3	-	$C_{26}H_{30}O_5$	M+H-H2O	↑	-
		Nimbaflavone	3	-	$C_{26}H_{30}O_5$	M+H-H2O	↑	-
		Abyssinone V 4'-methyl ether	3	-	$C_{26}H_{30}O_5$	M+H-H2O	↑	-
		Nitidulin	3	-	$C_{26}H_{30}O_5$	M+H-H2O	↑	-
604,352	4,65	Nidulanin A	3	-	$C_{34}H_{45}N5O_5$	M+H	↑	-

Supplementary Table 3: Metabolites that discriminate between control (C) and fed with dried citrus pulp (T) groups on serum metabolomics profile (all identification levels) and the corresponding mz (mass-to-charge-ratio), Rt (retention time), LI (level of identification), InChI Key, formula, adduct, FC (fold change of mass peak calculated as the ratio of signal intensity response in T group samples to that in the C group samples: ↑ upregulated (FC>1), downregulated (FC<1)), and Class (class).

**mz**	**RT (min)**	**Putative metabolites**	**LI**	**InChIKey**	**Formula**	**Adduct**	**FC**	**Class**
142,05	1,04	2-Hydroxymethylclavam	2	CRVXTEJTLQIKDI-XINAWCOVSA-N	C6H9NO3	M-H	↑	Alkaloids
		N-Acrylylglycine methyl ester	3	-	C6H9NO3	M-H	↑	-
		6-Oxopiperidine-2-carboxylic acid	2	FZXCPFJMYOQZCA-UHFFFAOYSA-N	C6H9NO3	M-H	↑	Carboxylic acids and derivatives
		5-ethyl-5-methyl-2,4-oxazolidinedione	2	MGHNWMRNFGYJKE-UHFFFAOYSA-N	C6H9NO3	M-H	↑	Azolidines
		Acrylamide-acrylic acid resin	2	RNIHAPSVIGPAFF-UHFFFAOYSA-N	C6H9NO3	M-H	↑	Carboxylic acids and derivatives
		Trimethadione	2	IRYJRGCIQBGHIV-UHFFFAOYSA-N	C6H9NO3	M-H	↑	Azolidines
		Vinylacetylglycine	2	UKISAGFGRDHYFO-UHFFFAOYSA-N	C6H9NO3	M-H	↑	Carboxylic acids and derivatives
195,05	0,73	L-Gulonate	3	RGHNJXZEOKUKBD-QTBDOELSSA-N	C6H12O7	M-H	↑	-
239,17	7,51	Dihydro-alpha-ionone	3	JHJCHCSUEGPIGE-LBPRGKRZSA-N	C13H22O	M+FA-H	↓	-
		Theaspirane	3	GYUZHTWCNKINPY-UHFFFAOYSA-N	C13H22O	M+FA-H	↓	-
		5-Isopropyl-2-(2-methylpropyl)-2-cyclohexen-1-one	3	RRVUWHZNUXPZBP-UHFFFAOYSA-N	C13H22O	M+FA-H	↓	-
		(E)-2-(2-Octenyl)cyclopentanone	3	QHEUOYOHVATZEB-VOTSOKGWSA-N	C13H22O	M+FA-H	↓	-

		4,7-Megastigmadien-9-ol	3	PWDOJWCZWKWKSE-BQYQJAHWSA-N	$C_{13}H_{22}O$	M+FA-H	↓	-
		Octahydro-6-isopropyl-2(1H)-naphthalenone	3	HEKJOMVJRYMUDB-UHFFFAOYSA-N	$C_{13}H_{22}O$	M+FA-H	↓	-
		beta-Ionol	3	CNOPDZWOYFOHGN-BQYQJAHWSA-N	$C_{13}H_{22}O$	M+FA-H	↓	-
		Geranylacetone	3	HNZUNIKWNYHEJJ-XFXZXTDPSA-N	$C_{13}H_{22}O$	M+FA-H	↓	-
		4-(2,6,6-Trimethyl-1-cyclohexen-1-yl)-2-butanone	3	QJJDNZGPQDGNDX-UHFFFAOYSA-N	$C_{13}H_{22}O$	M+FA-H	↓	-
		(+/-)-[R-(E)]-5-Isopropyl-8-methylnona-6,8-dien-2-one	3	PQDRXUSSKFWCFA-SOFGYWHQSA-N	$C_{13}H_{22}O$	M+FA-H	↓	-
		(3S,6E)-Nerolidol	3	JIFKIUVSUFVKTE-RSKUSDAESA-N	$C_{13}H_{22}O$	M+FA-H	↓	-
		7,8-Dehydro-3,4-dihydro-beta-ionol	3	WPGXAVCJKGSOBD-UHFFFAOYSA-N	$C_{13}H_{22}O$	M+FA-H	↓	-
		6,10-Dimethyl-5(E),9-undecadien-2-one	3	HNZUNIKWNYHEJJ-FMIVXFBMSA-N	$C_{13}H_{22}O$	M+FA-H	↓	-
		L-Menthyl acetoacetate	3	QSVQIPXQOCAWHP-UHTWSYAYSA-N	$C_{14}H_{24}O_3$	M-H	↓	-
		7-oxo-11E-Tetradecenoic acid	3	WPGZVCKWXQHYGF-ONEGZZNKSA-N	$C_{14}H_{24}O_3$	M-H	↓	-
		7-oxo-11Z-Tetradecenoic acid	3	WPGZVCKWXQHYGF-ARJAWSKDSA-N	$C_{14}H_{24}O_3$	M-H	↓	-
		(1S,2S)-3-oxo-2-pentyl-cyclopentanebutanoic acid	3	MQWNXLZVXAQZES-RYUDHWBXSA-N	$C_{14}H_{24}O_3$	M-H	↓	-
		(1R,2R)-3-oxo-2-pentyl-cyclopentanebutanoic acid	3	MQWNXLZVXAQZES-VXGBXAGGSA-N	$C_{14}H_{24}O_3$	M-H	↓	-

245,05	5,36	[3-(4-methoxyphenyl)propoxy]sulfonic acid	2	LZRUJECSWOAXOK-UHFFFAOYSA-N	C10H14O5S	M-H	↓	Ether
249,02	5,88	4,4'-Sulfonyldiphenol (Diphone C)	2	VPWNQTHUCYMVMZ-UHFFFAOYSA-N	C12H10O4S	M-H	↑	Phenols
257,18	7,24	DIBUTYL ADIPATE	3	XTJFFFGAUHQWII-UHFFFAOYSA-N	C14H26O4	M-H	↑	-
		1-Hydroxypropan-2-yl 2-isopropyl-5-methylcyclohexyl carbonate	3	ILYLFTONJARHOX-UHFFFAOYSA-N	C14H26O4	M-H	↑	-
		Diethyl decanedioate	3	ONKUXPIBXRRIDU-UHFFFAOYSA-N	C14H26O4	M-H	↑	-
		Tetradecanedioic acid	3	HQHCYKULIHKCEB-UHFFFAOYSA-N	C14H26O4	M-H	↑	-
		2-Hydroxypropyl 2-isopropyl-5-methylcyclohexyl carbonate	3	FLYJSXDJKBHQAU-UHFFFAOYSA-N	C14H26O4	M-H	↑	-
		Diisobutyl adipate	3	RDOFJDLLWVCMRU-UHFFFAOYSA-N	C14H26O4	M-H	↑	-
		Menthol propylene glycol carbonate	3	-	C14H26O4	M-H	↑	-
		2-methyl-tridecanedioic acid	3	BNDKRNYIWRVFJZ-UHFFFAOYSA-N	C14H26O4	M-H	↑	-
		3-methyl-tridecanedioic acid	3	XGGBLUZXXAOZCR-UHFFFAOYSA-N	C14H26O4	M-H	↑	-
		4-methyl-tridecanedioic acid	3	CBWZQKMGWFWYMF-UHFFFAOYSA-N	C14H26O4	M-H	↑	-
363,17	5,21	-	3	-	C16H22N4O3	M+FA-H	↑	-
391,2	6,01	(3S,4S)-3-hydroxytetradecane-1,3,4-tricarboxylic acid	3	QFOFNCNFUGQWTO-DYVFJYSZSA-N	C17H30O7	M+FA-H	↑	-

		(6RS)-22-oxo-23,24,25,26,27-pentanorvitamin D3 6,19-sulfur dioxide adduct / (6RS)-22-oxo-23,24,25,26,27-pentanorcholecalciferol 6,19-sulfur dioxide adduct	3	QPAPCSSCGKZVEI-RMZXUFQGSA-N	C22H32O4S	M-H	↑	-
		sevral Petides	3	-	C19H28N4O5	M-H	↑	-
		several Petides	3	-	C19H28N4O5	M-H	↑	-
417,21	6,39	9-Hydroxy-7-megastigmen-3-one glucoside	2	NLPBBNGDXNHAJP-AATRIKPKSA-N	C19H32O7	M+FA-H	↑	Fatty acyls
		Blumenol C glucoside	2	NYLNHNDMNOPWAZ-UHFFFAOYSA-N	C19H32O7	M+FA-H	↑	Carbohydrate derivative
419,23	6,78	several peptides	3	-	C15H30N6O5	M+FA-H	↑	-
		Octyl 2-O-alpha-L-fucopyranosyl-alpha-D-galactopyranoside	3	-	C20H38O10	M-H-H2O	↑	-
		Tsangane L 3-glucoside	3	UJRMJTIXXKZFGB-UHFFFAOYSA-N	C19H34O7	M+FA-H	↑	-
		Sclerotiotide F	3	KPPYRCDQUDIYQD-CAYKZORVSA-N	C21H32N4O5	M-H	↑	-
439,2	6,4	Pteroside Z	2	QFXWNTWJLHHEKX-UHFFFAOYSA-N	C21H30O7	M+FA-H	↑	Carbohydrate derivative
		Secoeremopetasitolide B	2	YEMKZDPASIYASW-YFHOEESVSA-N	C21H30O7	M+FA-H	↑	Terpene lactones
444,27	6,62	Uoamine A	2	FYOBRHSIFOGQKX-XROOOXQSSA-N	C22H41NO3S	M+FA-H	↑	Alkaloids
	7,2	CAY10412	3	-	C25H36O2S	M+FA-H	↑	-

	7,2	Atractyloside C	3	SJMJJDCFAGFDRH-SZDWJZJPSA-N	C21H36O7	M+FA-H	↑	-
	7,2	Sclerotiotide G	3	PECOIDLLRXSGKX-KFVQFUFLSA-N	C23H36N4O6	M-H-H2O	↑	-
447,26	7,08	N-Acetyl-leu-leu-tyr-amide	2	XKHMCCFBVQTJKS-UHFFFAOYSA-N	C23H36N4O5	M-H	↑	Peptide
		Sclerotiotide	2	DMCVGIKLKNPCAA-HIDOQYIDSA-N	C23H38N4O6	M-H-H2O	↑	Oligopeptides
459,22	6,04	Tentoxin	3	SIIRBDOFKDACOK-LFXZBHHUSA-N	C22H30N4O4	M+FA-H	↑	-
		several peptides	3	-	C22H30N4O4	M+FA-H	↑	-
477,1	5,28	Alliumoside A	3	VTDBDVABTGGRMO-UHFFFAOYSA-N	C22H22O12	M-H	↑	-
		Hesperetin 7-O-glucuronide	3	NEAWXAXVQDDFJL-PLEVBHNASA-N	C22H22O12	M-H	↑	-
		Pedaliin	3	WLDSVYQTJXGHOT-UHFFFAOYSA-N	C22H22O12	M-H	↑	-
		Estragonoside	3	HOQOGRHHARLRBG-UHFFFAOYSA-N	C22H22O12	M-H	↑	-
		1,3,5,8-Tetrahydroxy-6-methoxy-2-methylanthraquinone 8-O-b-D-glucoside	3	VAYMARBXYQUXAO-UHFFFAOYSA-N	C22H22O12	M-H	↑	-
		6-Methoxyluteolin 3'-glucoside	3	VYKCLUAGIMQEER-UHFFFAOYSA-N	C22H22O12	M-H	↑	-
		8-Hydroxyluteolin 4'-methyl ether 8-glucoside	3	JNHIUGKYXOYDMR-UHFFFAOYSA-N	C22H22O12	M-H	↑	-
		Isorhamnetin 7-glucoside	3	YCUNOXSUHVGZRI-UHFFFAOYSA-N	C22H22O12	M-H	↑	-

		Isorhamnetin 3-galactoside	3	CQLRUIIRRZYHHS-UHFFFAOYSA-N	C22H22O12	M-H	↑	-
		Azaleatin 3-glucoside	3	HJOBXPHWOUTSLV-UHFFFAOYSA-N	C22H22O12	M-H	↑	-
		Nepitrin	3	DMXHXBGUNHLMQO-IWLDQSELSA-N	C22H22O12	M-H	↑	-
		2-O-(4-Hydroxycinnamoyl)-1-O-galloyl-beta-D-glucopyranoside	3	SWCFDHFIUQJCLJ-UTCJRWHESA-N	C22H22O12	M-H	↑	-
		Hesperetin 3'-O-glucuronide	3	PJAUEKWZQWLQSU-WDXLFLMVSA-N	C22H22O12	M-H	↑	-
		Pollenin B	3	ATOXWOVZPMMCKB-UHFFFAOYSA-N	C22H22O12	M-H	↑	-
		Eupafolin 4'-glucoside	3	FKEFURJFBYTFMP-UHFFFAOYSA-N	C22H22O12	M-H	↑	-
		Tamarixetin 3-galactoside	3	JXASPPWQHFOWPL-OJZCZCLUSA-N	C22H22O12	M-H	↑	-
		8-C-Rhamnosyleuropetin	3	RCOZJKAJHKSREE-RZJMHOTASA-N	C22H22O12	M-H	↑	-
		8-Hydroxyluteolin 3'-methyl ether 7-glucoside	3	NNISLAFAXFMPCJ-PHBXNUNNSA-N	C22H22O12	M-H	↑	-
		Tamarixin	3	JXASPPWQHFOWPL-VZIAGGLWSA-N	C22H22O12	M-H	↑	-
		Europetin 3-rhamnoside	3	SCNKDAJBBGDFOB-YKGSUCIHSA-N	C22H22O12	M-H	↑	-
		Eupatolitin 3-apioside	3	OEAMGGHFQBSAKZ-LKXRKSRJSA-N	C22H22O12	M-H	↑	-
		Ranupenin 3-rhamnoside	3	PTUBUBVQXWVBPI-ISGYKXNISA-N	C22H22O12	M-H	↑	-
		Quercetin 3-methyl ether 5-glucoside	3	WAJZHIMAJHLZQB-BPVUJVISSA-N	C22H22O12	M-H	↑	-

		Quercetin 3-methyl ether 7-galactoside	3	LKXBGSZMRNJAST-XZJFLLRHSA-N	C22H22O12	M-H	↑	-
		Quercetin 3-methyl ether 7-glucoside	3	LKXBGSZMRNJAST-BPVUJVISSA-N	C22H22O12	M-H	↑	-
		Quercetin 3-methyl ether 3'-glucoside	3	VUGZODBTVLLVFQ-XZJFLLRHSA-N	C22H22O12	M-H	↑	-
		Quercetin 3-methyl ether 4'-glucoside	3	PCBCGLHWIGZJQI-UMTAUPKRSA-N	C22H22O12	M-H	↑	-
		6-Methoxyluteolin 7-glucoside	3	DMXHXBGUNHLMQO-PHBXNUNNSA-N	C22H22O12	M-H	↑	-
		6-Hydroxyluteolin 7-methyl ether 6-glucoside	3	WLDSVYQTJXGHOT-CIIGYETHSA-N	C22H22O12	M-H	↑	-
		Laricitrin 3-rhamnoside	3	UFWGANQPWYXUQI-YKGSUCIHSA-N	C22H22O12	M-H	↑	-
		6-Hydroxyluteolin 7-methyl ether 6-galactoside	3	WLDSVYQTJXGHOT-UIJDMCKQSA-N	C22H22O12	M-H	↑	-
		Syringetin 3-xyloside	3	YIPNLIAJDSNFQM-KFAWZMLGSA-N	C22H22O12	M-H	↑	-
		Nepetin 4'-glucoside	3	FKEFURJFBYTFMP-PHBXNUNNSA-N	C22H22O12	M-H	↑	-
		Mearnsitrin	3	NAQNISJXKDSYJD-KAWLKDMASA-N	C22H22O12	M-H	↑	-
		5,2',4',5'-Tetrahydroxy-7-methoxy-4-phenylcoumarin 5-O-glucoside	3	LKCRMORFQZVSHE-AKJGCJRBSA-N	C22H22O12	M-H	↑	-
		Annulatin 7-rhamnoside	3	LRKFQPXWFNUAOK-XVXUAGGESA-N	C22H22O12	M-H	↑	-
		Myricetin 3,4'-dimethyl ether 3'-xyloside	3	OEFVQTLECRIMST-ODCKNITQSA-N	C22H22O12	M-H	↑	-

		Azaleatin 3-galactoside	3	HJOBXPHWOUTSLV-LAHUMABZSA-N	$C_{22}H_{22}O_{12}$	M-H	↑	-
		Myricetin 5-methyl ether 3-rhamnoside	3	LPVCLSBWFKENQA-YKGSUCIHSA-N	$C_{22}H_{22}O_{12}$	M-H	↑	-
		6-Methoxykaempferol 3-galactoside	3	PMKDGKVUENNUGX-OAAUPYCUSA-N	$C_{22}H_{22}O_{12}$	M-H	↑	-
		6-Methoxykaempferol 3-glucoside	3	PMKDGKVUENNUGX-ZQAUCRDDSA-N	$C_{22}H_{22}O_{12}$	M-H	↑	-
		6-Hydroxykaempferol 3-methyl ether 6-glucoside	3	RCCHIJJQDUZNBN-ILDYPZKUSA-N	$C_{22}H_{22}O_{12}$	M-H	↑	-
		6-Hydroxykaempferol 3-methyl ether 7-glucoside	3	DKJSFZFROHHPOX-KWJWMMQSSA-N	$C_{22}H_{22}O_{12}$	M-H	↑	-
		6-Methoxykaempferol 7-glucoside	3	RVCQJMUKRAYQKT-QLSHPEDTSA-N	$C_{22}H_{22}O_{12}$	M-H	↑	-
		Sexangularetin 3-galactoside	3	LZSGYESPQHEVBU-HGKHRKFHSA-N	$C_{22}H_{22}O_{12}$	M-H	↑	-
		6-Hydroxykaempferol 4'-methyl ether 7-glucoside	3	ZJEAJHNKCFZPND-MFEGBOHYSA-N	$C_{22}H_{22}O_{12}$	M-H	↑	-
		Sexangularetin 3-glucoside	3	LZSGYESPQHEVBU-LUBREZGKSA-N	$C_{22}H_{22}O_{12}$	M-H	↑	-
		Keyakinin B	3	PBYPIFDEBZJDAM-MVMWKIHSSA-N	$C_{22}H_{22}O_{12}$	M-H	↑	-
		Rhamnetin 3-galactoside	3	PHEWILLIAJUBQE-AJSGNWPESA-N	$C_{22}H_{22}O_{12}$	M-H	↑	-
		Rhamnetin 3-glucoside	3	PHEWILLIAJUBQE-IFGFCIQDSA-N	$C_{22}H_{22}O_{12}$	M-H	↑	-
		Rhamnetin 5-glucoside	3	CLYDDTSYSNAITP-GCXDDFLKSA-N	$C_{22}H_{22}O_{12}$	M-H	↑	-
		Herbacetin 7-methyl ether 3-glucoside	3	ATOXWOVZPMMCKB-FUVQAQHDSA-N	$C_{22}H_{22}O_{12}$	M-H	↑	-

		Isorhamnetin 4'-glucoside	3	VTDBDVABTGGRMO-IXQPCOCWSA-N	C22H22O12	M-H	↑	-
		Isorhamnetin 3-glucoside	3	CQLRUIIRRZYHHS-RQMGYXQJSA-N	C22H22O12	M-H	↑	-
		Tricetin 3'-methyl ether 7-glucoside	3	ASGQQIRHJIOWQM-AKJGCJRBSA-N	C22H22O12	M-H	↑	-
		Tamarixetin 7-glucoside	3	HGZHKWZIXPLKLY-GCXDDFLKSA-N	C22H22O12	M-H	↑	-
		Patuletin 3-rhamnoside	3	HVLWQYAHDXFNSN-ISGYKXNISA-N	C22H22O12	M-H	↑	-
479,26	5,71	Several peptides	3	-	C21H34N6O4	M+FA-H	↑	-
		6'-Hydroxysimvastatin	3	-	C25H38O6	M+FA-H	↑	-
		3''-Hydroxysimvastatin	3	-	C25H38O6	M+FA-H	↑	-
		3'-Hydroxysimvastatin	3	-	C25H38O6	M+FA-H	↑	-
		6'-Hydroxymethylsimvastatin	3	-	C25H38O6	M+FA-H	↑	-
		(12S,15S)-15-O-Demethyl-10,29-dideoxy-11,12-dihydro-striatin C	3	FTZQOXPOWUWXJV-UHFFFAOYSA-N	C25H38O6	M+FA-H	↑	-
		Erinacine C	3	DMPGFSQMXITJPT-UHFFFAOYSA-N	C25H38O6	M+FA-H	↑	-
120,08	0,767	isoindoline	3	GWVMLCQWXVFZCN	C8H9N	M+H	↓	-
		para-Cresidine	3	WXWCDTXEKCVRRO-UHFFFAOYSA-N	C8H11NO	M+H-H2O	↓	-
		1-(p-Hydroxyphenyl)ethylamine	3	CDQPLIAKRDYOCB-UHFFFAOYSA-N	C8H11NO	M+H-H2O	↓	-

		Methyridine	3	QRBVCAWHUSTDOT-UHFFFAOYSA-N	C8H11NO	M+H-H2O	↓	-
		Phenylethanolamine	3	-	C8H11NO	M+H-H2O	↓	-
		p-Phenetidine	3	-	C8H11NO	M+H-H2O	↓	-
		(R)-(-)-2-Phenylglycinol	3	-	C8H11NO	M+H-H2O	↓	-
		meta-Cresidine	3	CDGNLUSBENXDGG-UHFFFAOYSA-N	C8H11NO	M+H-H2O	↓	-
		2-Hydroxyphenethylamine	3	ULSIYEODSMZIPX-UHFFFAOYSA-N	C8H11NO	M+H-H2O	↓	-
		(±)-2-(2-Furanyl)pyrrolidine	3	LIFJPSLQRGQNMM-UHFFFAOYSA-N	C8H11NO	M+H-H2O	↓	-
		4-Hydroxy-2,6-dimethylaniline	3	GCWYXRHXGLFVFE-UHFFFAOYSA-N	C8H11NO	M+H-H2O	↓	-
		2-Acetyl-1-ethylpyrrole	3	HQADRFRTIALOCB-UHFFFAOYSA-N	C8H11NO	M+H-H2O	↓	-
		1,2,3,4,5,6-Hexahydro-7H-cyclopenta[b]pyridin-7-one	3	JOAMMKKZSZEDHD-UHFFFAOYSA-N	C8H11NO	M+H-H2O	↓	-
		m-Tyramine	3	GHFGJTVYMNRGBY-UHFFFAOYSA-N	C8H11NO	M+H-H2O	↓	-
		Tyramine	3	DZGWFCGJZKJUFP-UHFFFAOYSA-N	C8H11NO	M+H-H2O	↓	-
		Dimethylaniline-N-oxide	3	LKQUDAOAMBKKQW-UHFFFAOYSA-N	C8H11NO	M+H-H2O	↓	-
129,07	0,656	(R)-3-Ureidoisobutyrate	2	PHENTZNALBMCQD-GSVOUGTGSA-N	C5H10N2O3	M+H-H2O	↑	Ureas
		Isoglutamine	2	AEFLONBTGZFSGQ-UHFFFAOYSA-N	C5H10N2O3	M+H-H2O	↑	Amino acids

		Glycylalanine	2	-	C5H10N2O3	M+H-H2O	↑	Oligopeptides
		Ureidoisobutyric acid	3	PHENTZNALBMCQD-UHFFFAOYSA-N	C5H10N2O3	M+H-H2O	↑	-
		L-Glutamine	2	ZDXPYRJPNDTMRX-VKHMYHEASA-N	C5H10N2O3	M+H-H2O	↑	Amino acids
		D-Glutamine	2	ZDXPYRJPNDTMRX-GSVOUGTGSA-N	C5H10N2O3	M+H-H2O	↑	Amino acids
		Alanylglycine	2	CXISPYVYMQWFLE-UHFFFAOYSA-N	C5H10N2O3	M+H-H2O	↑	Oligopeptides
130,09	0,679	-	3	-	C6H11NO2	M+H	↑	-
		N-Hydroxy-L-isoleucine	3	YEGAKLYOVHUQIJ-WHFBIAKZSA-N	C6H13NO3	M+H-H2O	↑	-
		(4S)-4-Hydroxy-L-isoleucine	3	OSCCDBFHNMXNME-YUPRTTJUSA-N	C6H13NO3	M+H-H2O	↑	-
		2-amino-6-hydroxyhexanoic acid	3	OLUWXTFAPJJWPL-UHFFFAOYSA-N	C6H13NO3	M+H-H2O	↑	-
		Fagomine	3	YZNNBIPIQWYLDM-HSUXUTPPSA-N	C6H13NO3	M+H-H2O	↑	-
		(2R,3R,4R)-2-Amino-4-hydroxy-3-methylpentanoic acid	3	OSCCDBFHNMXNME-UHFFFAOYSA-N	C6H13NO3	M+H-H2O	↑	-
		N-(2-Hydroxyethyl)-morpholine N-oxide	3	WTPGFRPRHSDKNE-UHFFFAOYSA-N	C6H13NO3	M+H-H2O	↑	-
144,1	0,676	Proline betaine (stachydrine)	2	CMUNUTVVOOHQPW-LURJTMIESA-N	C7H13NO2	M+H	↑	Proline and derivatives
		L-2-Amino-3-methylenehexanoic acid	2	ZJMPUKNPMYTOOX-UHFFFAOYSA-N	C7H13NO2	M+H	↑	Alpha amino acids
		3beta,6beta-Dihydroxynortropane	2	MVUIPZFMWQBRCM-UHFFFAOYSA-N	C7H13NO2	M+H	↑	Tropane alkaloids

146,12	0,682	2-amino-heptanoic acid	3	-	C7H15NO2	M+H	↑	-
		3-Dehydroxycarnitine	3	JHPNVNIEXXLNTR-UHFFFAOYSA-N	C7H15NO2	M+H	↑	-
171,12	0,76	1,6,7-Trimethylnaphthalene	3	JBXULKRNHAQMAS-UHFFFAOYSA-N	C13H14	M+H	↓	-
		1,4,5-Trimethyl-naphtalene	3	FSAWRQYDMHSDRN-UHFFFAOYSA-N	C13H14	M+H	↓	-
		Aethusin	3	OUUDSRYJNWAHPD-FIXGFOPZSA-N	C13H14	M+H	↓	-
261,14	3,466	gamma-Glutamylleucine	2	MYFMARDICOWMQP-YUMQZZPRSA-N	C11H20N2O5	M+H	↑	Oligopeptides
261,18	0,666	Carisoprodol	3	OFZCIYFFPZCNJE-UHFFFAOYSA-N	C12H24N2O4	M+H	↑	-
275,17	0,668	Lysyl-Glutamine	2	OAPNERBWQWUPTI-UHFFFAOYSA-N	C11H22N4O4	M+H	↑	Oligopeptides
		Lysyl-Gamma-glutamate	2	MEFNTMKESSGLSP-UHFFFAOYSA-N	C11H22N4O4	M+H	↑	Oligopeptides
287,2	0,678	N-Acetyl-leucyl-leucine	3	SQGBTKIDTKOYMS-UHFFFAOYSA-N	C14H26N2O4	M+H	↑	-
625,24	5,698	Penicillin F	3	QRLCJUNAKLMRGP-ZTWGYATJSA-N	C14H20N2O4S	2M+H	↓	-
		PS-6	3	SUMQHCXEWHRKGG-MWLCHTKSSA-N	C14H20N2O4S	2M+H	↓	-
		Met Tyr	3	-	C14H20N2O4S	2M+H	↓	-
		Tyr Met	3	-	C14H20N2O4S	2M+H	↓	-
		Tyrosyl-Methionine	3	KYPMKDGKAYQCHO-UHFFFAOYSA-N	C14H20N2O4S	2M+H	↓	-
		Methionyl-Tyrosine	3	PESQCPHRXOFIPX-UHFFFAOYSA-N	C14H20N2O4S	2M+H	↓	-

		1-Naphtaldehyde	3	PRDPBCXFXCOEBM-UHFFFAOYSA-N	$C_{22}H_{16}O_2$	2M+H	↓	-
